# Thermodynamic Stability of the Icosahedral Quasicrystal i‑ScZn: A Reassessment of the Sc–Zn Phase Diagram

**DOI:** 10.1021/acsomega.6c04466

**Published:** 2026-08-21

**Authors:** Michael Sannemo-Targama, Yu-Chin Huang, Girma Gebresenbut, Akash Khansili, Roman Lucrezi, Ulrich Häussermann

**Affiliations:** † Department of Chemistry, 7675 Stockholm University, 106 91 Stockholm, Sweden; ‡ Department of Chemistry, Ångström Laboratory, 8097 Uppsala University, 751 21 Uppsala, Sweden; § Department of Physics, AlbaNova University Center, Stockholm University, 106 91 Stockholm, Sweden

## Abstract

The thermodynamic stability of quasicrystals (QCs) and their relationship to compositionally adjacent phases in intermetallic phase diagrams remain incompletely understood. We examined the icosahedral QC i-ScZn (Sc_12_Zn_88_), which is embedded in the Sc–Zn system between the cubic 1/1 approximant crystal (AC) phase ScZn_6_ (Sc_14.3_Zn_85.7_) and the ScZn_12_ phase (Sc_7.7_Zn_92.3_) adopting the tetragonal ThMn_12_ structure. Single crystalline (or single grain) samples of ScZn_6_, Sc_12_Zn_88_, and ScZn_12_ were obtained by solution growth from Sc_*x*_Zn_100–*x*_ melts under appropriate composition and temperature conditions. Variable-temperature powder X-ray diffraction and thermal analysis experiments reveal that as-cast Sc_12_Zn_88_ undergoes annealing-induced decomposition into Sc_12+*x*_Zn_88–*x*_ (*x* ≈ 1) and ScZn_12_, indicating the existence of a temperature-dependent homogeneity range of the i-ScZn phase. Upon heating, i-ScZn decomposes peritectically at approximately 625 °C into ScZn_6_ and liquid. Interpretation of the phase equilibria is complicated by pronounced kinetic undercooling of approximately 30 °C associated with the crystallization of ScZn_12_, which decomposes on heating at 505 °C into i-ScZn and liquid, but crystallizes only upon cooling to 475 °C. The present results support prior suggestions of an enthalpy-driven stabilization of i-ScZn, while establishing Sc_12+*x*_Zn_88–*x*_ (*x* ≈ 1) as the thermodynamically stable QC composition at low temperatures.

## Graphical abstract

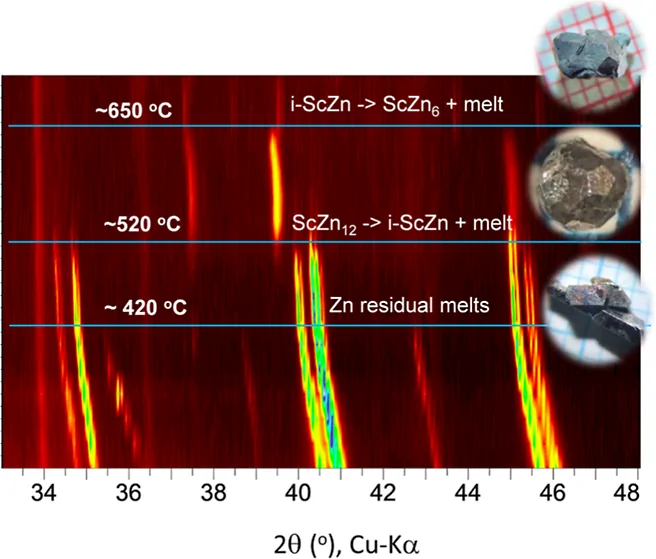

## 1 Introduction

The discovery of thermodynamically stable icosahedral quasicrystals (QCs) in the binary intermetallic systems RE-Cd (RE = Y, Gd–Yb), Ca–Cd, and Sc–Zn marked a significant advance in the understanding of the structural principles and physical properties of aperiodically ordered solids. In particular, it enabled the growth of large mm scale grains or single domain specimens from the melt under slow cooling rates, thereby facilitating comprehensive investigations of composition, atomic structures, and physical properties.^1–5^ These binary Cd- and Zn-based QCs established a distinct third class of icosahedral quasicrystals, now commonly referred to as Tsai-type QCs. Since their initial discovery, Tsai-type QCs have developed into the most prolific family of QCs—spanning several dozens of representatives (both stable and metastable)—by the creation of ternary variants upon substituting Cd partially for Mg or Zn (and for Zn-based QCs, substituting Zn partially for, e.g., Au),^6^ or completely by binary mixtures of late transition metals and early p-elements.^7^ The magnetic properties of RE containing Tsai-type QCs have recently attracted considerable attention because of expectations about unique magnetic states based on the long-range ordering of spins in a quasiperiodic structure.^8–11^

The structure of Tsai-type QCs corresponds to an arrangement of condensed and interpenetrating rhombic triacontahedron (RTH) clusters, also called Tsai clusters.^3,12^ The Tsai cluster itself consists of a series of concentric polyhedral shells of which the outermost RTH is followed by an icosidodecahedron (IDD), an icosahedron (ICO), a dodecahedron (DOD), and a tetrahedron (TET) at the center (Figure 1a). The alkaline-earth or RE component (M) of the QC is segregated in the ICO shell, whereas TET breaks the icosahedral symmetry of a Tsai cluster and is typically orientationally (dynamically or statically) disordered.^13^ The Tsai cluster is also the building block of quasicrystal approximant crystals (ACs), which are intermetallic phases with a composition and atomic arrangement similar to that of QCs but possessing periodic crystal structures. The simple 1/1 AC has a body-centered cubic unit cell (space group *Im*3̅), which hosts two Tsai clusters (Figure 1b).^14^ The higher order 2/1 AC (space group *Pa*3̅, Figure 1c) is more structurally similar to a QC.^15^ Again, the primary building block is the Tsai cluster, but in addition there is a bicapped double Friauf polyhedron (DFP), Figure 1d, with the shape of an acute rhombohedron which fills the interstitial space between RTHs.^11,15^ In the QC structure, RTH and DFP units are aperiodically arranged, yet RTHs follow the same linkage principle as in the AC structures. Interestingly, Cd-based Tsai-type QCs appear compositionally flexible, and two branches are discussed: branch I with composition MCd_5.7_ (M_15_Cd_85_) found with M = Yb, Ca and branch II with composition MCd_7.28–7.88_ (M_12_Cd_88_–M_11.3_Cd_88.7_) found with M = Y, Gd, Tb, ..., Tm.^3,16,17^ Whereas for branch I, the ICO positions and the centers of the DFPs are exclusively occupied by M, for branch II, these positions are partially occupied by Cd.

**Figure 1:**
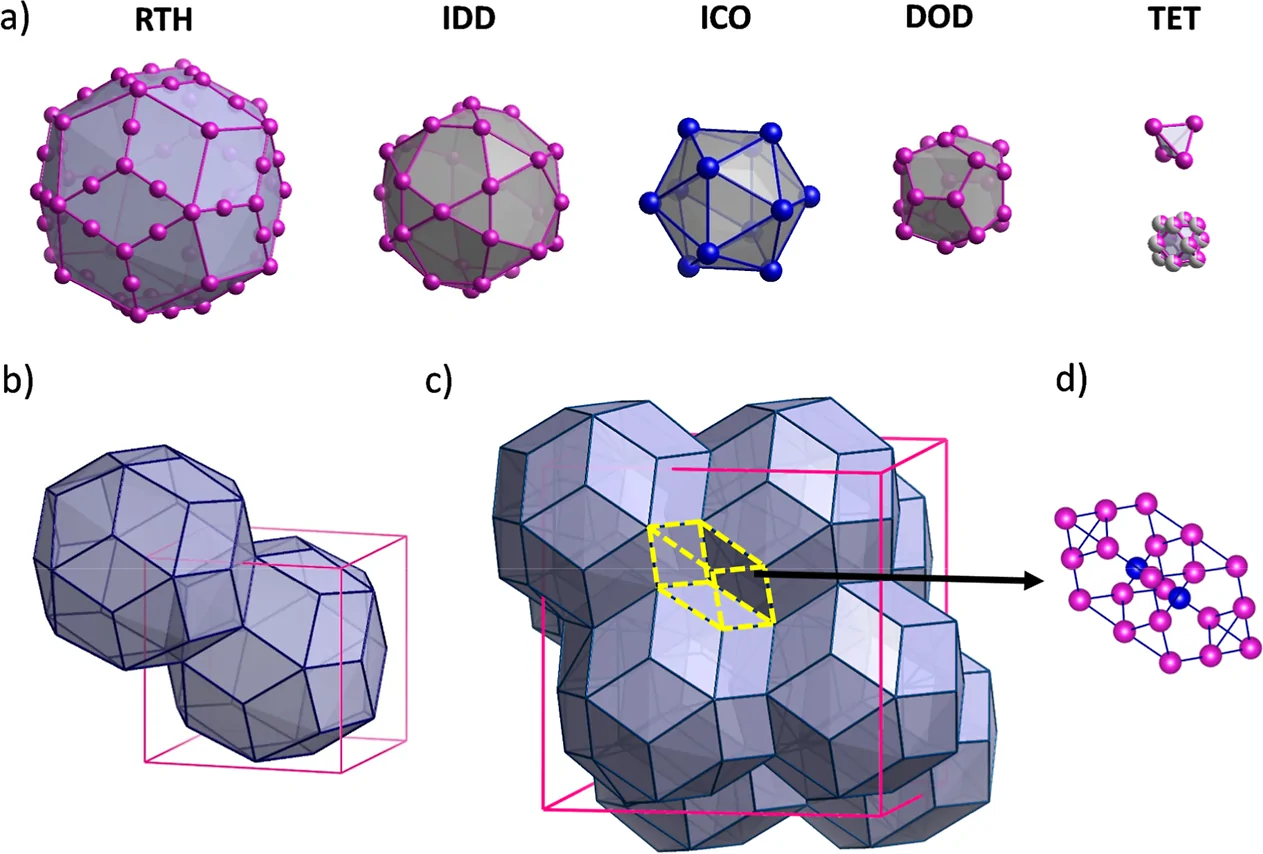
(a) Atomic decoration of the concentric shells constituting a Tsai cluster. The rhombic triacontahedron (RTH) is the outermost shell, followed by an icosidodecahedron (IDD), an icosahedron (ICO), a dodecahedron (DOD), and a (disordered) tetrahedron (TET). (b,c) Arrangement of RTHs in 1/1 and 2/1 approximant crystals (ACs), respectively. The latter reveals a second building block, an acute rhombohedron, which corresponds to a double Friauf polyhedron (DFP), shown in (d), and which is also found in the QC structure. Pink spheres denote Zn or Cd atoms and blue spheres alkaline earth (AE) or rare-earth (RE) metal atoms. In type I Tsai-type QCs, the positions of the ICO shell and DFP centers are exclusively occupied by AE/RE atoms. In type II QCs, these positions are mixed occupied with 20–30% Zn/Cd.

Although the existence of thermodynamically stable QCs (i.e., representing equilibrium phases in intermetallic phase diagrams) is now well established, the conditions governing their stabilization—particularly the relative contributions of enthalpic vs entropic terms—remain a matter of ongoing debate.^18–21^ Unique to aperiodic solids is the presence of additional degrees of freedom, namely phason fluctuations, which may contribute significantly to the entropy beyond conventional configurational, vibrational, and electronic terms.^22–24^ However, experimental identification and quantitative assessment of phason-related contributions via excess heat capacity measurements have proven challenging.^25–29^ At low temperatures, static phason strain is expected to increase the enthalpy while simultaneously introducing configurational entropy contributions.

Binary Tsai-type QCs are especially well suited for fundamental investigations into QC stability owing to the relative simplicity of binary phase diagrams. Among known binary QC-forming systems, the Sc–Zn phase diagram (Figure 2a) is distinguished because the QC (Sc_12_Zn_88_, with branch II composition^4^) is not the most Zn-rich phase but is instead embedded between the periodic crystal phases ScZn_12_ (with the tetragonal ThMn_12_ structure, Figure 2c) and ScZn_6_ (a 1/1 AC, cf. Figure 1b). A recent computational investigation of the Sc–Zn system by Baek et al.—based on density functional theory and elegantly tackling the challenges associated with modeling aperiodic structures—revealed thermodynamic stability of Sc_12_Zn_88_ at 0 K, thus strongly indicating that stabilization of an aperiodic QC phase can arise exclusively from enthalpic contributions.^30^ Furthermore, these authors proposed that solidification of Sc_12_Zn_88_ from the melt is kinetically limited by nucleation processes, and introduced a combined thermodynamic–kinetic phase diagram to rationalize both equilibrium stability and crystallization behavior of the Zn-rich phases ScZn_6_, Sc_12_Zn_88_, and ScZn_12_.

**Figure 2:**
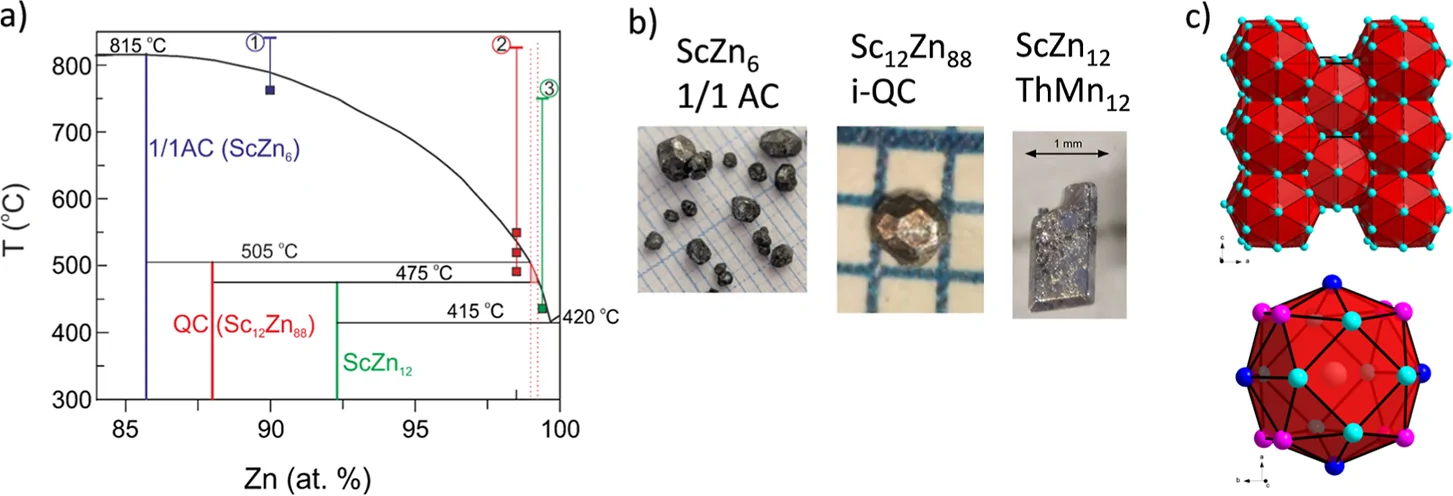
(a) Zinc-rich part of the Sc–Zn phase diagram according to Canfield et al.^4^ and based on the one introduced by Palenzona et al.^44^ The extremely small wedge for accessing Sc_12_Zn_88_ QC phase from solution growth is indicated in red and by the broken vertical lines. Applied conditions for solution growth synthesis of “as-cast” samples of ScZn_6_, Sc_12_Zn_88_, and ScZn_12_ are indicated by solid vertical lines where squares mark extraction temperatures. (b) “As-cast” sample specimens ScZn_6_ (AC), Sc_12_Zn_88_ (QC), and ScZn_12_ as obtained from solution growth. Note the fullerene (truncated icosahedron)-shaped morphology of the QC grain. (c) ThMn_12_ structure of ScZn_12_. The lower figure shows the 20-atom coordination polyhedron around Sc. The three different Zn positions are distinguished by different colors.

In the present work, we revisited the Sc–Zn system with the aim of critically assessing the thermodynamic stability of Sc_12_Zn_88_ through a comprehensive investigation of its phase relations with the adjacent compounds ScZn_6_ and ScZn_12_. Particular attention was given to experimental conditions as previous studies were hampered by the high volatility of Zn at temperatures above 650 °C.

## 2 Methods

### 2.1 Solution Growth Synthesis of ScZn_12_, Sc_12_Zn_88_, and ScZn_6_ Specimens

Granules of Sc (99.99%) and Zn (99.9999%) purchased from ChemPUR were used as starting materials. Reaction mixtures with batch sizes 2–5 g were placed in “Canfield Crucible sets (CCS)”^31^ with 1.7 mL dimension, which in turn were sealed inside stainless-steel tubes by welding under an Ar atmosphere. The CCS setup allows for effective separation of crystalline phase from excess melt at elevated temperatures by centrifugation. The synthesis of ScZn_12_ employed a reaction mixture with composition Sc_0.75_Zn_99.25_, which was heated to 750 °C and annealed at this temperature for 3 h to ensure a homogeneous melt. Subsequently, the temperature was lowered to 600 °C at a rate of 50 °C/h, followed by slow cooling from 600 to 425 °C at a rate of 2 °C/h. Synthesis targeting Sc_12_Zn_88_ employed reaction mixtures with nominal compositions Sc_1.5_Zn_98.5_–Sc_2.5_Zn_97.5_, which were heated to 820 °C and after homogenization (3 h) cooled to 600 °C (75 °C/h) followed by slow cooling (2 °C/h) to temperatures in a range 490–590 °C. The synthesis of ScZn_6_ employed a reaction mixture with the nominal composition Sc_10_Zn_90_, which was heated to 850 °C and after homogenization (3 h) cooled to 800 °C (50 °C/h) followed by slow cooling (5 °C/h) to 750 °C. After reaching the final temperature, the syntheses were terminated by isothermally centrifuging off excess melt at 4000 rpm. Temperatures were monitored with an auxiliary type K thermocouple placed close to the sample position in the furnace.

### 2.2 Thermal Analysis Experiments

For the various thermal analysis experiments, a Netzsch STA 449 F1 Jupiter thermal analysis apparatus was employed. Samples were mostly placed in a sapphire crucible (5 mm diameter), which was subsequently seal welded in a stainless-steel capsule (5.5 mm diameter, 5 mm height) in an Ar filled glovebox. Experiments were performed in the presence of an oxygen getter (Zr metal) and using a continuous Ar gas flow (5N, ∼40 mL/min). Typically, samples were analyzed by PXRD after thermal investigation. Differential scanning calorimetry (DSC) measurements were performed for as-cast ScZn_12_, Sc_12_Zn_88_, and ScZn_6_. These investigations used 10–20 mg sized grain specimens as samples. Heating/cooling cycles were performed to temperatures between 600 and 850 °C and using heating/cooling rates of 10 °C/min. In order to extract liquidus temperatures, DSC measurements were also performed for elemental mixtures with compositions Sc_2.5_Zn_97.5_, Sc_5_Zn_95_, Sc_10_Zn_90_, and Sc_12_Zn_88_. For these studies, 100 mg sized samples were enclosed in Ta capsules, see below, and heated to 850 °C to produce a homogeneous melt. Liquidus temperatures were extracted upon cooling (10 °C/min).

In addition, annealing experiments were performed in the well controllable environment of the Jupiter STA apparatus. These concerned Sc_12_Zn_88_ samples (in the form of grain specimens or powdered, 20–50 mg) which were treated at temperatures between 200 and 400 °C for extended periods of time (4–12 h). Further annealing experiments targeted the formation of Sc_12_Zn_88_ from stoichiometric mixtures of ScZn_6_ and ScZn_12_ (molar ratio 3.5:1) by solid-state reaction. 50–100 mg of finely ground and carefully mixed precursors were pressed into 3 mm diameter pellets, which were annealed at 400 , 450, and 490 °C for 24 and 48 h. Samples for annealing experiments were placed in sapphire crucibles and the seal welded in stainless-steel capsules.

The thermal analysis studies revealed a high volatility of Zn at temperatures above ca. 650 °C. This posed challenges for liquidus line studies requiring temperatures above 800 °C to achieve homogeneous melts. To avoid empty sample capsule volume, we employed thin-walled Ta capsules (OD 3.8 mm, wall-thickness: 0.25 mm), crimped to a length corresponding to the volume of the sample, and welded shut. Due to the substantial vapor pressure of Zn, pressure buildup would occasionally cause failure of the capsule, which was detected as a sudden onset of weight-loss monitored by the simultaneously acquired thermogravimetric (TG) data during the measurement. For the most Zn-rich mixtures, repeated measurements were necessary until one was completed without capsule failure. However, despite our best efforts, we were not able to reliably prevent Zn volatilization.

### 2.3 PXRD Measurements

Powder X-ray diffraction (PXRD) measurements were performed with a Bruker D8 Powder diffractometer using either monochromated Cu Kα_1_ (λ = 1.540598 Å or Cu Kα (λ = 1.5418 Å)) radiation. Standard measurements employed θ–2θ diffraction (Bragg–Brentano) geometry and powder patterns were collected using a zero-background specimen holder, with the two-theta angle ranging from 20° to 90° and an exposure time of 0.75 s per step at 0.015° per measurement step at room temperature. The data were analyzed using HighScore Plus 3.0e software from PANalytical.^32^ For variable temperature PXRD experiments, finely ground samples were packed and sealed in 0.3 mm ID fused silica capillaries (ca. 20–30 mm sample height) in an Ar filled glovebox. The sealing was performed by melting the open end and rolling it against a copper hearth, using the arc of an arc-welder as a heat source. The measurements employed Bruker’s TC transmission chamber and data were collected between 2θ = 25°–50° with step size 0.017° per second. The heating rate was 20 °C per min and data collection (ca. 25 min) was started 1 min after having reached the target temperature. ScZn_12_ samples were measured up to 725 °C and the Sc_12_Zn_88_ sample up to 600 °C. Target temperatures were corrected against a calibration measurement using NaCl and utilizing the well-known thermal expansion of NaCl,^33^ see Figure S8.

Quasilattice parameters (*a*_ico_) of the QC samples were extracted from PXRD patterns using the six-dimensional indexing method as described by Elser.^34^ For an icosahedral QC, the *d*-spacing of a reflection depends on the quasilattice parameter, the six-dimensional indices, and a set of six basis vectors spanning parallel space (*E*_∥_). These basis vectors are permutations of the vectors pointing from the center of an icosahedron to its vertices, where *a*_ico_ scales the magnitude of these basis vectors as such

$$d=\frac{a_{ico}}{\left|\sum_{i=1}^{6}\left(e_{{∥}_{i}}n_{i}\right)\right|}$$

A manual least-squares analysis was performed by taking the sum of squared differences between observed and calculated values of *Q* ${\left(Q_{obs}-Q_{calc}\right)}^{2}$ , where $Q=\frac{2\pi }{d}$ , and minimizing it by adjusting *a*_ico_.

### 2.4 SEM Imaging and EDX Analysis

Scanning electron microscopy (SEM) and energy-dispersive spectroscopy (EDX) analyses were performed with a JEOL JSM IT-800 equipped with a Schottky-type field emission gun, a retractable 6 segment annular backscattered electron detector, and a high-resolution Si-drift detector for detecting EDX X-rays. For consistency, all measurements were performed with a working distance of 10 mm and acceleration voltage of 15 kV. Probe current was adjusted for a dead time of 15%. EDX spectra were collected from 60 s point scans and ∼30 of them were collected from which compositions were averaged and standard deviations calculated. Pieces or fragments of sample specimens with sizes ∼50–100 μm were prepared for analysis by cross-section (CP) polishing. Samples were mounted on Al stubs with hot-melt glue and their surface preleveled by initial wet grinding with 15 μm (1200P) SiC paper-backed abrasive followed by progressively finer lapping (4 μm, 2 μm, 1 μm) with diamond lapping suspension on a cloth lapping disc. The CP was performed with a JEOL SM-09010 cross-section polisher under Ar, using a beam voltage of 5.3 kV and a 20 h duration. Powder samples were prepared by mounting in phenolic resin and automatically polishing using the same sequence of progressively finer grits as used for the preprocessing of the CP samples. To eliminate effects from charge-up and lowering of the Duane–Hunt limit,^35^ all samples were sputtered with gold with a JEOL JFC-1200 fine coater (10 mA at 0.6 mbar for 60 s).

### 2.5 DFT Calculations

DFT relaxations and enthalpy calculations were performed via the Quantum ESPRESSO (QE) package,^36–38^ employing scalar-relativistic optimized norm-conserving Vanderbilt (ONCV) pseudopotentials from the SG15 ONCV library^39,40^ in combination with the PBE^41^ exchange–correlation functional. The kinetic energy cutoff for the wave functions was set to 120 Ry, the convergence threshold for the electronic self-consistency to 10^–10^ Ry, and a Methfessel–Paxton smearing^42^ value of 0.01 Ry was used for the metallic occupation numbers. We recalculated the formation energies of the known conventional (3D) crystalline phases of the Sc–Zn system: CsCl-type ScZn (2 atoms/unit cell), AlB_2_-type ScZn_2_ (3 atoms/unit cell), hexagonal Sc_13_Zn_58_ (Gd_13_Zn_58_-type, 142 atoms/unit cell), 1/1 AC ScZn_6_ (monoclinic ordered low-temperature structure, 168 atoms/unit cell), and tetragonal ScZn_12_ (ThMn_12_ structure, 13 atoms/unit cell in the primitive body-centered tetragonal cell). The **k**-grid spacing for the large ScZn_6_ and Sc_13_Zn_58_ structures was set to 0.020 × 2π Å^–1^ and to 0.012 × 2π Å^–1^ for all other structures. The **k**-grid was doubled along each reciprocal lattice vector and the energy cutoff was 300 Ry. The unit cells were relaxed below a threshold of 0.1 kbar for the stress tensor components and below 10^–6^ Ry/*a*_0_ for all atomic force components. These settings provide a numerical accuracy for the formation energy with respect to the hcp elemental structures of Sc and Zn on the order of 1 meV/atom, as validated against reference calculations with a doubled k-grid along each reciprocal lattice vector and an energy cutoff of 300 Ry.

## 3 Results and Discussion

### 3.1 Synthesis and Formation of i-ScZn Phase

Solution growth represents a powerful synthetic route to thermodynamically stable phases that are difficult to obtain from conventional high-temperature synthesis methods.^43^ The method has been successfully applied for the growth and isolation of large (mm)-sized grains of binary QCs in the RE-Cd and Sc–Zn systems.^4,5^ The Sc–Zn phase diagram (Figure 2a) was originally reported by Palenzona et al.^44^ and subsequently refined by Canfield et al., who identified the i-ScZn (Sc_12_Zn_88_) QC phase^4^ and corrected the composition of the 1/1 AC phase from Sc_3_Zn_17_ to ScZn_6_, consistent with the finding of Lin and Corbett.^45^ Guided initially by this phase diagram, we produced bulk samples of ScZn_6_, i-ScZn, and ScZn_12_ by solution growth (Figures 2b and S1); these samples are hereafter referred to as “as-cast”. Obviously, ScZn_6_ can be obtained over a wide range of melt compositions (∼86–∼95 at. % Zn). In the present work, a reaction mixture Sc_10_Zn_90_ was used. In contrast, the reported phase diagram suggests that the regions of exposed liquidus for accessing i-ScZn and ScZn_12_ are extremely narrow, namely 0.8–1.0 at. % Sc at 475–505 °C for i-ScZn and 0.3–0.8 at. % Sc at 415–475 °C for ScZn_12_. While ScZn_12_ was obtained at 0.75 at. % Sc, in agreement with the phase diagram, phase-pure i-ScZn could be synthesized at significantly higher Sc concentrations (1–2 at. %) and at temperatures up to 550 °C, well above the reported peritectic temperature (∼505 °C), as seen in Figure 3. These observations indicate that the peritectic temperature of i-ScZn and the shape of the liquidus line deviate from the established diagram. We stress that reliable synthesis results depended critically on carefully weighed Sc–Zn reaction mixtures, large batch sizes (i.e., minimal empty space in the CCS setup) to minimize the effect of Zn volatility, and accurate temperature measurement via a thermocouple positioned in close proximity of the sample in the CCS setup.

**Figure 3:**
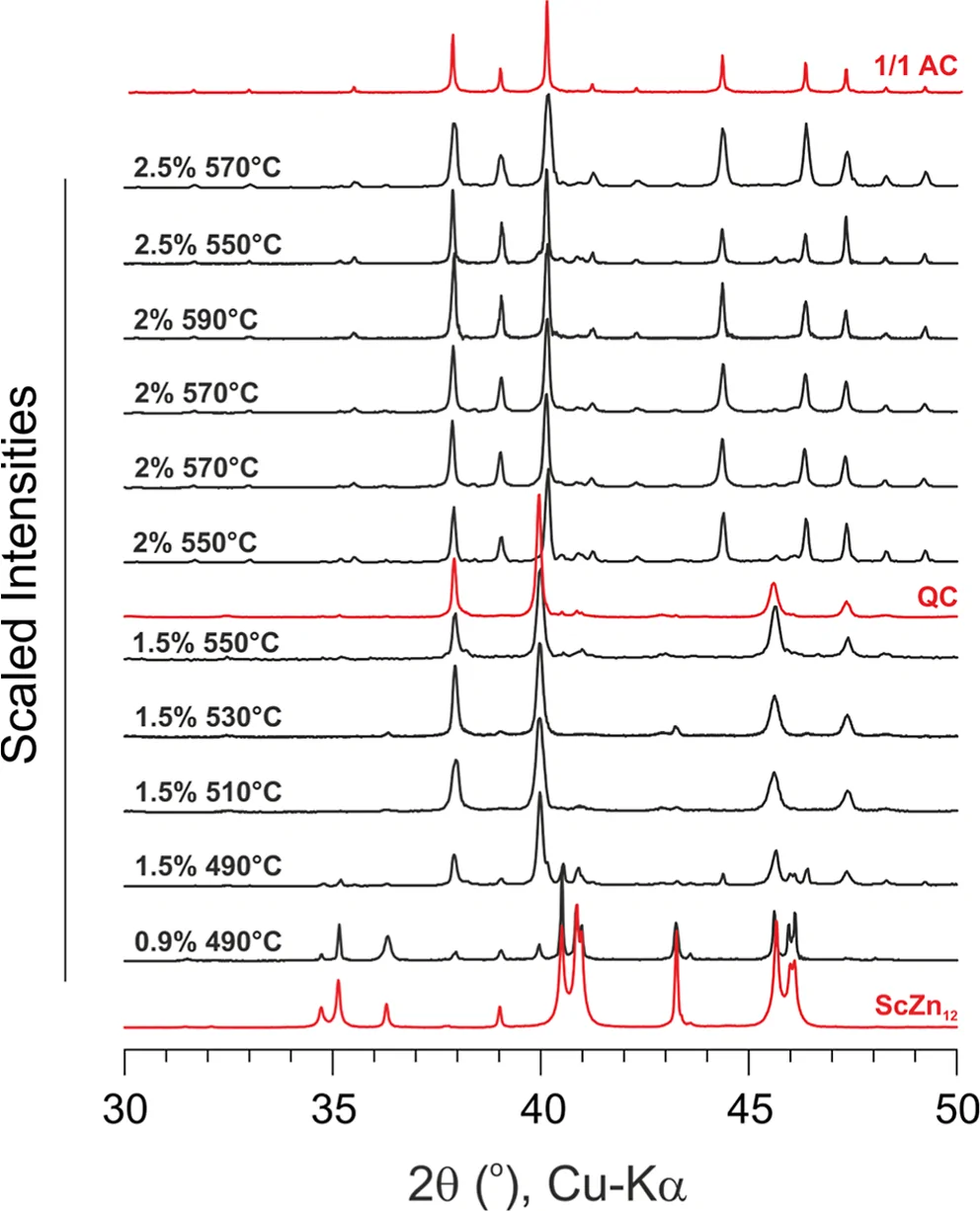
Compilation of PXRD patterns of products from solution growth experiments targeting Sc_12_Zn_88_. % refers to at. % Sc in the applied reaction mixtures, temperatures refer to extraction temperatures. The patterns of pure 1/1 AC, i-ScZn (QC) and ScZn_12_ samples are added as references (red color).

Previous studies reported the extraction of as-cast i-ScZn from the melt at approximately 480 °C.^4,16^ EDX analysis of our sample obtained at 490 °C indicates a slightly Sc-enriched composition of Sc_12.1(2)_Zn_87.9(2)_ (Figure S2) compared to the earlier report.^4^ For samples extracted at higher temperatures, the Sc content seems to be increased (Table 1, Figures S3 and S4). Nevertheless, for consistency, the as-cast quasicrystalline phase will hereafter be denoted Sc_12_Zn_88_.

**Table 1:** Compositions and Quasilattice Parameters of i-ScZn Samples

sample source	melt separation/synthesis temperature (°C)	EDX composition *x*_Sc_	quasilattice parameter *a*_ico_ (Å)
solution growth	490	12.1(2)	5.017(6)
	510	12.2(2)	5.017(6)
	530	12.5(2)	5.016(7)
	550	12.3(1)	5.028(8)
	∼480 Canfield et al. (2010)^4^	12.0(3)	5.017(3)
	∼480 Yamada et al. (2016)^16^		5.021(3)
3.5 ScZn_6_ + ScZn_12_	490	12.5(2)	5.023(6)

As an alternative to solution growth, one may attempt the synthesis of i-ScZn from the peritectic reaction AC + L ↔ QC. However, reactions AC + L ↔ QC are rarely observed since they require extremely fine-grained AC precursors (<1 μm) for overcoming the sluggish kinetics of QC formation.^46^ Cooling of a melt Sc_7.7_Zn_92.3_ (nominally ScZn_12_) from 800 to 490 °C and subsequent extraction produces large mm sized ScZn_6_ AC crystals (Figure S5). Admixed to these AC crystals is a small fraction of 20–100 μm sized pentagonal dodecahedron-shaped QC grains, partly separated and partly attached to AC surfaces. Judging from the largely intact AC surfaces, these grains do not appear to have formed from a peritectic reaction AC + L ↔ i-ScZn but precipitated from the melt—below the peritectic line—consistent with an increase in the solid fraction upon cooling. A reliable compositional analysis of these grains was not feasible.

The Sc–Zn system also suggests the formation of QC from a solid-state reaction between ScZn_6_ and ScZn_12_ (3.5 ScZn_6_ + ScZn_12_ ↔ 4.5 ScZn_7.33_, equivalently expressed as 0.654 Sc_14.3_Zn_85.7_ + 0.346 Sc_7.7_Zn_92.3_ ↔ Sc_12_Zn_88_). Here, we demonstrate this synthesis pathway (Figure 4): Finely ground and pelletized stoichiometric mixtures of ScZn_6_ and ScZn_12_, subjected to isothermal annealing for 48 h, exhibit partial QC formation at 450 °C and complete conversion at 490 °C. Notably, stoichiometric mixtures always showed the complete consumption of ScZn_6_ reactant whereas a smaller fraction of unreacted ScZn_12_ remained. Compositional analysis of the product indicates a slightly, but significantly, higher Sc content compared to as-cast samples, yielding Sc_12.45(15)_Zn_87.55(15)_ (Sc_12+*x*_Zn_88–*x*_), Figure S6. At the same time, we point out a further inconsistency with the phase diagram shown in Figure 2a, which suggests that ScZn_12_ decomposes at 475 °C into Sc_12_Zn_88_ and melt, thereby potentially enabling completion of Sc_12+*x*_Zn_88–*x*_ phase by initiating an AC + L reaction. We will show below that upon heating, ScZn_12_ is stable up to about 505 °C. In contrast, when cooling a composition “ScZn_12_” (Sc_7.7_Zn_92.3_) from high temperatures, crystalline ScZn_12_ does not form above about 475 °C.

**Figure 4:**
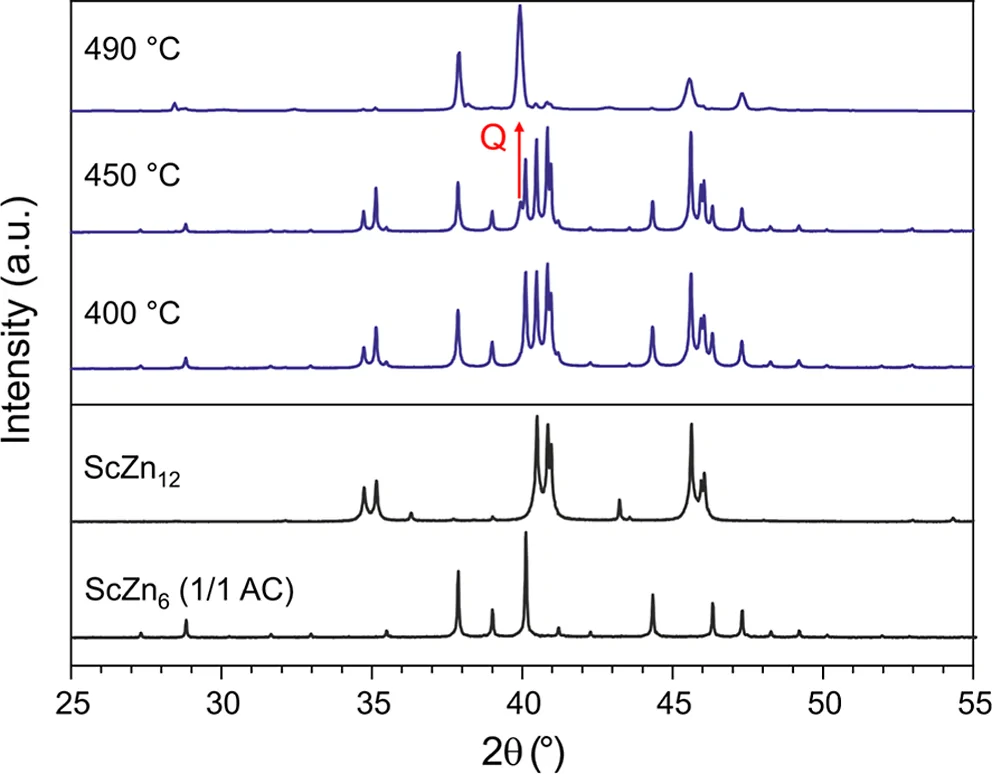
PXRD patterns of products during 48 h solid-state reactions 3.5 ScZn_6_ + ScZn_12_ at 400 °C, 450 °C, and 490 °C. “Q” accompanied by a red arrow marks the most prominent i-ScZn reflection in the 450 °C pattern. The bottom panels show the PXRD patterns of the precursors ScZn_6_ and ScZn_12_.

The compositions and quasilattice parameters of our various i-SnZn samples (Sc_12_Zn_88_ from solution growth with melt separation at temperatures between 490 and 550 °C and Sc_12+*x*_Zn_88–*x*_ from the solid-state reaction) are summarized in Table 1 and compared to previously reported values for solution grown samples. From this compilation, it can be inferred that the small compositional variations revealed by EDX analysis do not result in significant changes in the lattice parameter.

### 3.2 Stability of Sc_12_Zn_88_ and Phase Relation to ScZn_12_ toward a Revised Sc–Zn Diagram

As a first step into reassessing the phase relations between AC, QC, and ScZn_12_, we performed DSC investigations on the as-cast samples. The results are compiled in Figure 5. The heating/cooling trace for ScZn_6_ (Figure 5a) shows melting/decomposition at around 820 °C, in agreement with the phase diagram. The heating trace reveals a small amount of Zn (or rather ScZn_12_–Zn eutectic) impurity, melting at 415 °C, which is attributed to residual flux on the surface of the specimen. In addition, there is a weak event at 505 °C, which we will comment on below. The PXRD pattern after the DSC heating/cooling cycle shows a ScZn_6_ AC phase and traces of hexagonal Sc_13_Zn_58_, Figure 5b. This observation highlights a general problem in the thermal analysis of Zn-rich Sc–Zn intermetallics, which is also described in Section 2.2: despite negligible mass loss indicated by TG, partial volatilization of Zn occurs within the sealed sample container and the resulting segregation of Zn from the bulk sample leads to subtle but significant compositional deviations.

**Figure 5:**
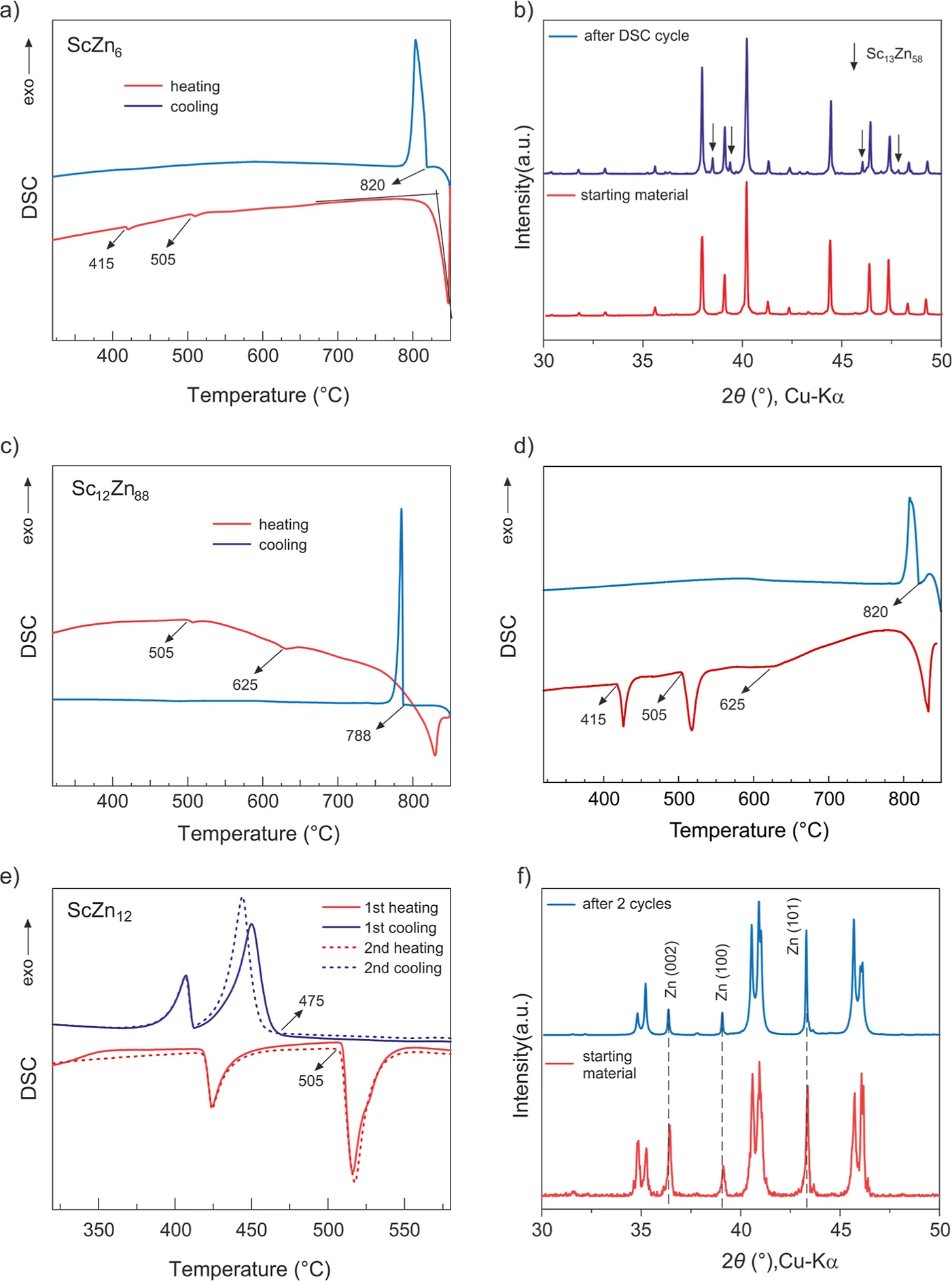
(a) DSC traces of a heating/cooling cycle for ScZn_6_. (b) PXRD patterns of ScZn_6_ before and after the DSC experiment. (c,d) DSC traces of a heating/cooling cycle for i-ScZn (Sc_12_Zn_88_). The sample employed for (d) contained a significant amount of ScZn_12_–Zn eutectic as impurity. (e) DSC traces of a two heating/cooling cycles for ScZn_12_. (f) PXRD patterns of ScZn_12_ before and after the DSC experiment.

Similarly, the Sc_12_Zn_88_ sample shows a weak event at 505 °C (Figure 5c). A comparable, though more pronounced, feature was previously reported by Canfield et al. and attributed to the peritectic decomposition of the Sc_12_Zn_88_ QC phase.^4^ The heating trace reveals a further weak event around 625 °C, followed by an event at ∼800 °C corresponding to the liquidus crossing. In Figure 5d, we show the DSC trace for a Sc_12_Zn_88_ specimen with a substantial amount of residual flux impurity, whose presence seems to drastically enhance the signal at 505 °C. As in Figure 5c, a weak feature near ∼625 °C is discernible, along with the liquidus crossing at ∼800 °C. Upon cooling, the corresponding event appears at ∼820 °C, which is very similar to the ScZn_6_ sample in Figure 5a, and indicative of crystallization of the AC phase. Post-DSC PXRD analysis of both samples (Figure 5c,d) reveals an exclusive presence of the ScZn_6_ AC phase, again indicating segregation of some Zn from the bulk.

Most conspicuous is the thermal behavior of ScZn_12_ specimens (which were almost always accompanied by various amounts of residual flux impurity). The heating trace shows melting of residual flux at 415 °C, followed by an event at around 505 °C (Figure 5e). Upon cooling, this transition is instead observed at 475 °C. A second heating/cooling cycle is virtually identical, reproducing this behavior (505 °C on heating, 475 °C on cooling). The PXRD pattern after a two-cycle DSC treatment appears identical to the pattern of the starting material (Figure 5f). Since in this experiment, the sample was only heated to 600 °C, abnormalities due to the volatility of Zn were not noticeable. This thermal hysteresis appears to be independent of experimental conditions, like heating/cooling rate (cf. Figure S7), container material, mass of sample, and amount of residual flux. As is outlined below, we attribute this behavior to kinetic undercooling of ScZn_12_ nucleation.

To gain better insights into the thermal behavior of Sc_12_Zn_88_ and ScZn_12_, we performed variable-*T* PXRD experiments, which are summarized in Figures 6 and 7. Upon heating of ScZn_12_ (Figure 6a,b), melting of residual flux (ScZn_12_–Zn eutectic) is recognized by the disappearance of Zn reflections between 411 and 460 °C and peritectic decomposition to QC phase is observed between 511 and 535 °C. Whereas the DSC experiments (cf. Figure 5e) suggest a fast process, QC phase formation in the (horizontally aligned) PXRD capillary is rather sluggish. A complete conversion of ScZn_12_ into i-ScZn + melt required annealing at 535–555 °C for several hours. Upon further heating, QC phase was seen stable (in the presence of melt) up to at least 613 °C (Figure 6a). At 662 °C, reflections from QC were absent and instead reflections from the AC phase were recognizable (cf. Figure S9). Because of the increased fraction of melt, the quality of the PXRD patterns deteriorate upon QC decomposition. Also, solid and melt segregate which leads to an inhomogeneous distribution of sample in the capillary. Consequently, the peritectic formation of ScZn_12_ from i-ScZn + melt upon cooling was not observed, Figure 6b.

**Figure 6:**
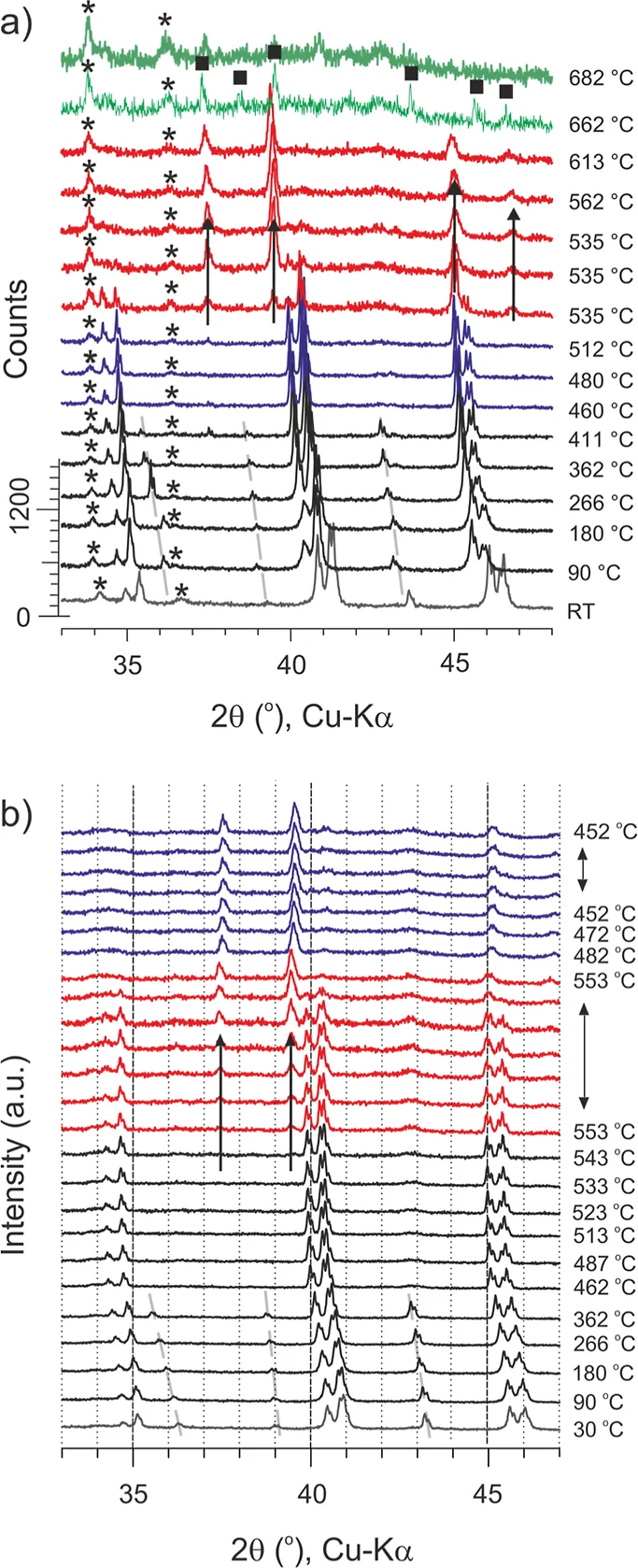
Variable-*T* PXRD measurements for ScZn_12_ (Cu Kα radiation (λ = 1.5418 Å)). (a) Heating from RT to 682 °C through two peritectic decomposition steps (cf. Figure S8). Asterisks mark reflections from the sample environment. Gray broken lines mark reflections from Zn impurity. Black squares denote reflections from the ScZn_6_ AC phase. (b) Heating from 40 to 552 °C and annealing at 552 °C for complete i-ScZn phase conversion followed by subsequent slow cooling to 373 °C. The formation of i-ScZn phase in (a,b) is indicated by arrows. Patterns are color-grouped to indicate transition regions or significant segments. Gray broken lines mark reflections from Zn impurity.

**Figure 7:**
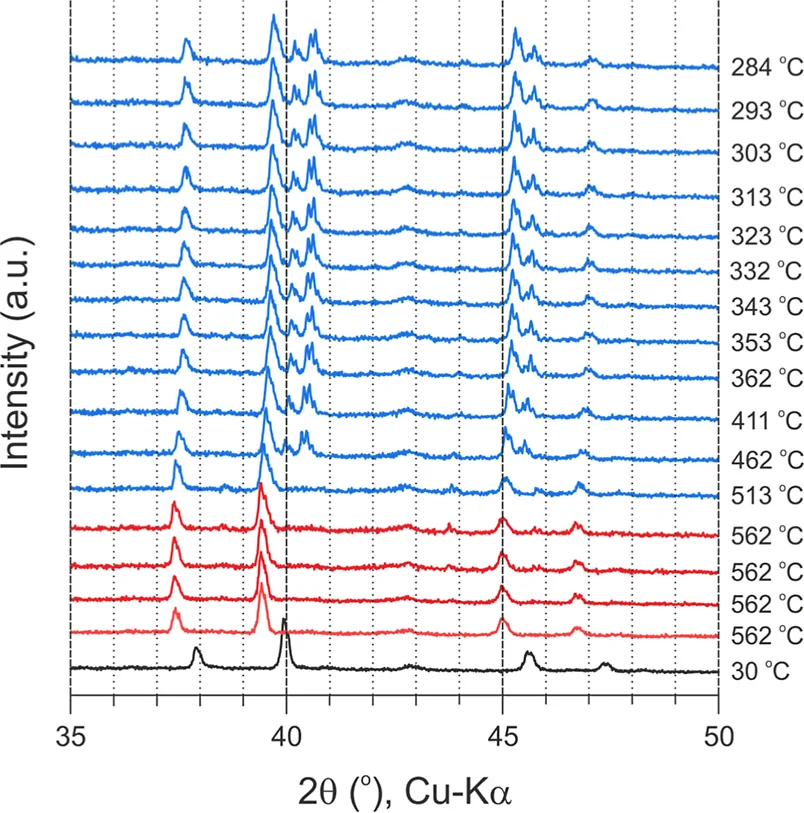
Variable-*T* PXRD measurement for i-ScZn (Sc_12_Zn_88_). Rapid heating (20 °C/min) from 30 °C (black pattern) to 562 °C is followed by annealing for 4 h (red patterns) and subsequent slow cooling to 285 °C (ca. 20 °C/h, blue patterns).

In the variable-*T* PXRD experiment for Sc_12_Zn_88_, the sample was first heated to 562 °C at 20 °C/min (over the course of ca. 30 min), annealed for 2 h and then slowly cooled, Figure 7. Upon heating and during annealing, there was no noticeable change in the diffraction patterns. However, upon cooling below 475 °C, ScZn_12_ reflections suddenly appeared, and their intensity remained unchanged during further cooling. This implies either the decomposition of Sc_12_Zn_88_ to Sc_12+*x*_Zn_88–*x*_ and ScZn_12_ is at around 475 °C or, and more likely, that Sc_12_Zn_88_ already decomposed during annealing at ∼ 560 °C to Sc_12+*x*_Zn_88–*x*_ and melt, and peritectic formation of ScZn_12_ takes place at around 475 °C upon cooling. Variations in the quasilattice parameter of i-ScZn during this experiment are shown in Figure S10. A detailed analysis of the *T*-dependence of the lattice parameter of i-ScZn is provided in Figure S11. However, from these data, it is not possible to infer an altered composition for i-ScZn at high temperatures, cf. Table 1.

The variable-*T* PXRD experiment shown in Figure 7 provides clear evidence that as-cast Sc_12_Zn_88_ obtained from solution growth is metastable. In support, annealing of finely ground material at 200–400 °C for 6 h produced ScZn_12_ at 300 and 400 °C, whereas (presumably for kinetic reasons) decomposition was not noticeable when grain specimens were annealed at 400 °C for 12 h, as seen in Figure S12. Together with the results of the synthesis experiments, cf. Table 1, this suggests a temperature-dependent homogeneity range for i-ScZn, Sc_12+*x*_Zn_88–*x*_, with a maximum *x* value ≈ 1 and extending toward Zn-rich compositions (*x* ≈ 0) around 500 °C. The notorious thermal event at ∼505 °C upon heating Sc_12_Zn_88_ is attributed to the peritectic decomposition of ScZn_12_, which itself originates from the prior low-temperature decomposition of metastable Sc_12_Zn_88_. The amount of ScZn_12_ formed depends on the heating rate and grain/particle size of Sc_12_Zn_88_. The presence of residual flux will increase substantially the amount of ScZn_12_ in the range 415–500 °C (cf. Figure 5d), likely accounting for earlier observations reported by Canfield et al.,^4^ see also Figure S13. The peritectic decomposition of i-ScZn near ∼625 °C manifests only as a very weak thermal event.

The unusual thermal behavior of ScZn_12_ may be rationalized in terms of kinetic undercooling: Upon heating, ScZn_12_ undergoes peritectic decomposition at ∼505 °C into i-ScZn and liquid. At this temperature, the equilibrium composition of i-ScZn is close to Sc_12_Zn_88_ (cf. Table 1). Upon cooling, however, reformation of ScZn_12_ is delayed until ∼475 °C owing to sluggish nucleation kinetics. We propose that the pronounced kinetic undercooling reflects a strong dependence of the nucleation barrier on the composition of the coexisting i-ScZn phase, with nucleation of ScZn_12_ becoming more favorable only after the quasicrystal composition has shifted toward higher Sc contents during cooling. The interpretation of our observations is summarized in the revised phase diagram presented in Figure 8 and Table 2. The liquidus line at low Sc concentrations was further constrained by DSC measurements (Figure S14) and is considered reliable down to 5 at. %. A comparison between the proposed liquidus and previously reported phase diagrams is provided in Figure S15.

**Figure 8:**
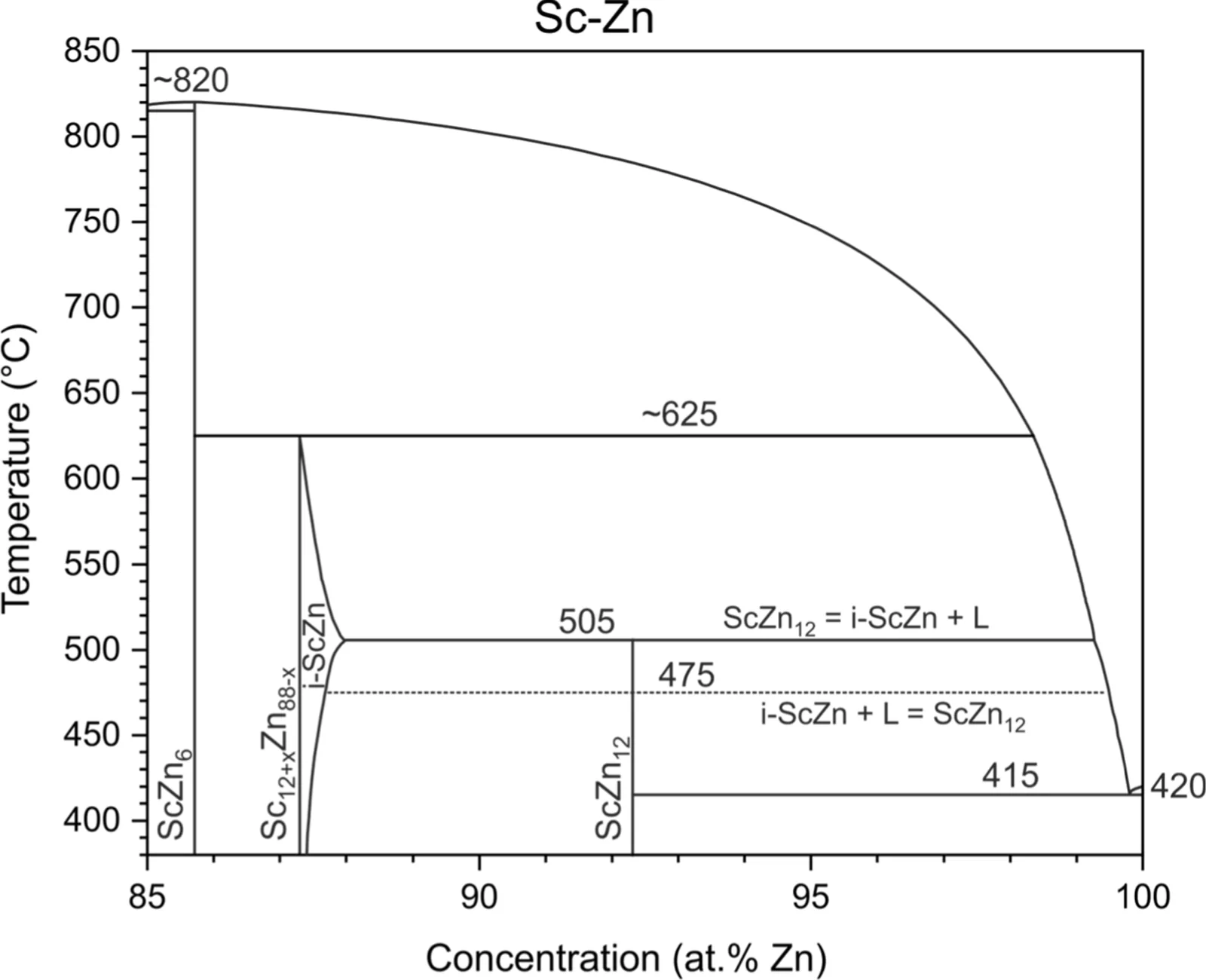
Proposed revision of the reported Sc–Zn phase diagram^4,44^ (cf. Figure 2a). The dotted horizontal line expresses kinetic undercooling associated with the crystallization of ScZn_12_.

**Table 2:** Overview of Phases in the Zn-Rich Part of the Sc–Zn System According to Figure 8

phase	conc. (at.% Sc)	formation reaction	type	trans. temp. (°C)	space group	type structure	lattice params. (Å)^a^
Zn	0	L → Zn	congruent	420	*P*6_3_/*mmc*	hcp	*a*: 2.6636(2)
							*c*: 4.9457(3)
ScZn_12_	7.7	L + i-ScZn → ScZn_12_	peritectic	505	*I*4/*mmm*	ThMn_12_	*a*: 8.800(2)
							*c*: 5.160(2)
i-ScZn	∼13	L + ScZn_6_ → i-ScZn	peritectic	∼625	*Pm*35̅	i-YbCd	*a*_ico_: 5.023(6)
ScZn_6_	14.3	L → ScZn_6_	congruent	820	*Im*3̅	YCd_6_	*a*: 13.8311(5)

a Lattice parameters refer to room temperature. Zn according to Nuss et al.,^47^ ScZn_12_ according to Sannemo-Targama et al.^48^ i-ScZn according to this work and referring to the product of the solid-state reaction ScZn_6_ + ScZn_12_ at 490 °C. ScZn_6_ according to Yamada et al.^49^

### 3.3 Thermochemical Considerations

First-principles DFT methods have been previously employed to assess the convex hull stability of Sc–Zn phases by calculating *T* = 0 K enthalpies (energies) of formation,^50^ and the Sc–Zn phase diagram is also included in the materials project.^51^ More recently, Baek et al. extended computational approaches to the aperiodic Sc_12_Zn_88_ by introducing the “nanocrystal size extrapolation” strategy.^30^ However, in all previous calculational works there seems to be some confusion with respect to the composition and structure of the stable periodic crystalline phases. This concerns especially the ScZn_6_ AC phase for which at room-temperature Zn_4_ tetrahedra at the Tsai cluster center (i.e., the TET shell, cf. Figure 1) are dynamically disordered.^49,52^ TET motion freezes at around 160 K upon which the cubic phase may undergo a phase transition into a monoclinic low-temperature structure or attain a statically disordered “glassy” state. The monoclinic phase^53,54^ should be considered as the ground state for ScZn_6_.

We recalculated the formation energies of the known Sc–Zn phases using their proper compositions and structures and added the value for Sc_12_Zn_88_ from Baek et al.^30^ Figure S13 compares the convex hull with that of previous calculations. An experimental standard formation enthalpy (referring to 298 K) is only known for ScZn.^55^ The reported value, −41.5 kJ/mol atom, is in reasonable agreement with the calculated ones,^30,50,51^ which scatter between −36 and −37 kJ/mol atom (referring to 0 K), cf. Figure S13. In Figure 9, we show the relevant Zn-rich region. Baek et al. did not consider the ordered monoclinic structure of ScZn_6_ but treated the AC with their nanocrystal size extrapolation method, analogous to the QC Sc_12_Zn_88_.^30^ The resulting structure would then approximate the disordered glassy state of ScZn_6_, which becomes situated slightly above the hull by 8 meV/atom (included in Figure 9 and Table 3). In contrast, considering the ordered monoclinic structure leads to a significant stabilization of ScZn_6_, and the AC phase ends up solidly on the hull. This, however, does not change the main result of Baek et al., namely, the *T* = 0 K convex hull stability of the QC Sc_12_Zn_88_. The stability of Sc_12_Zn_88_ pushes ScZn_12_ slightly above the hull (12 and 4 meV/atom for Baek et al. and our work, respectively (Table 3)).

**Figure 9:**
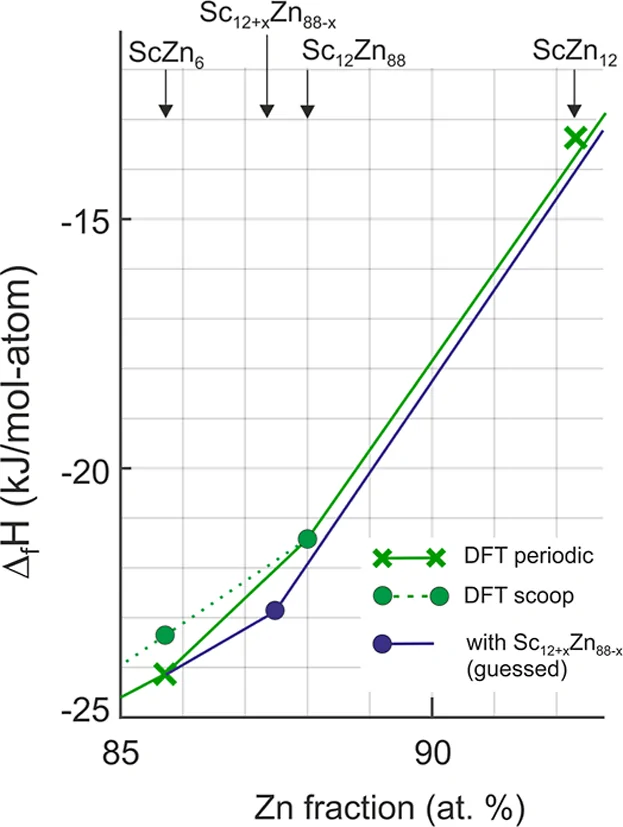
Convex hull stability of the most Zn-rich Sc–Zn phases. **Green:** DFT calculated *T* = 0 K formation energies, crosses refer to periodic structure, circles refer to disordered/aperiodic structures taken from Baek et al.,^30^ (“DFT scoop”). Broken and solid lines indicate the *T* = 0 K hull for disordered and ordered ScZn_6_, respectively. **Blue:** adding guessed Sc_12+*x*_Zn_88–*x*_.

**Table 3:** DFT Formation Enthalpies (Energies) Referring to *T* = 0 K^a^

phase	eV/atom	kJ/mol atom
ScZn_6_ – monocl.	–0.250	–24.14
ScZn_6_ – scoop	–0.242 (+0.008)	–23.35 (+0.79)
Sc_12_Zn_88_ – scoop	–0.222	–21.42
ScZn_12_	–0.139 (+0.004)	–13.37 (+0.37)
ScZn_12_–Baek	–0.130 (+0.012)	–12.54 (+1.19)

a The distances from the convex hull are indicated in parentheses.

To explain the experimentally observed phase separation tendency into Sc_12+*x*_Zn_88–*x*_ and ScZn_12_, one needs to assume that Sc_12_Zn_88_ is metastable, at least at temperatures below ∼400 °C, and put a value for the formation enthalpy of Sc_12+*x*_Zn_88–*x*_ (slightly) below the line between ScZn_6_ and Sc_12_Zn_88_. With this, ScZn_12_ would be positioned further above the convex hull (cf. Figure 9), indicating metastability at 0 K. We point to the possibility that ScZn_12_ is thermodynamically stable only at elevated temperatures. The ThMn_12_ structure for ScZn_12_ implies an unusually large (20-atom) coordination polyhedron for comparatively small Sc (cf. Figure 2c), resulting in a size-mismatched polyhedral framework.^48^ This situation could give rise to pronounced phonon anharmonicity, which may contribute decisively to vibrational (entropic) stabilization of ScZn_12_ at high temperatures. To rigorously assess thermodynamic stability relations, it will ultimately be necessary to determine the enthalpy and entropy of formation via calorimetric and heat capacity measurements, respectively, thereby enabling an experimentally grounded construction of the convex hull for the Sc–Zn system. A more detailed understanding of the thermodynamic properties of ScZn_12_ is considered essential for elucidating the formation and thermodynamic stability of i-ScZn at elevated temperatures.

## 4 Conclusion

The reinvestigation of the Zn-rich part of the Sc–Zn phase diagram led to the discovery that i-ScZn possesses a temperature-dependent homogeneity range Sc_12+*x*_Zn_88–*x*_ (0 < *x* < ≈1) and decomposes peritectically at around 625 °C, which is at considerably higher temperatures than previously reported (505 °C). As-cast Sc_12_Zn_88_ obtained by solution growth from Zn-rich melts at temperatures around 500 °C represents a metastable composition at room temperature. The compositional spread of Sc_12+*x*_Zn_88–*x*_ does not give rise to a significant variation in the lattice parameter. This can be attributed both to the small magnitude of the phase width (approximately 1 at. %) and to the similar effective atomic radii of Sc and Zn in polar Sc–Zn intermetallic compounds.^48^

Yamada et al. observed significant diffuse scattering in high-resolution synchrotron single-crystal X-ray diffraction patterns of Sc_12_Zn_88_ grains, with anisotropic distribution along the 3-fold direction.^17^ This was interpreted as resulting from phason fluctuations and Ishimasa suggested that Sc_12_Zn_88_,–now established as metastable at room temperature, is situated at the limit of periodic (3D) stability.^56^ A detailed understanding of the formation and thermodynamic stability of i-ScZn at elevated temperatures is still outstanding.

## Supplementary Material



## Notes

PXRD patterns and photographs of as-cast samples, SEM–EDX results for as-cast Sc_12_Zn_88_ (from solution growth) and Sc_12+*x*_Zn_88–*x*_ obtained from solid-state reaction between ScZn_6_ and ScZn_12_, *T* calibration for variable *T*-PXRD measurements and heat map plot highlighting the measurement of ScZn_12_, variation of the quasilattice parameter of i-ScZn with temperatures, PXRD patterns for Sc_12_Zn_88_ annealed between 200 and 400 °C, DSC heating/cooling cycles for ScZn_12_ with rates between 10 and 50 °C/min, complementary DSC experiments simulating previously reported results for Sc_12_Zn_88_ and for extracting the liquidus temperatures for 12, 10, 5, and 2.5 at. % Sc, comparison of previously reported liquidus lines with this work, and extended hull (50–100 at. % Zn) covering all Sc–Zn phases from DFT calculations (PDF)
