# Supplementary material for: Thermodynamic Stability of the Icosahedral Quasicrystal i‑ScZn: A Reassessment of the Sc–Zn Phase Diagram
Source: ACS Omega. 2026 Aug 21;11(34):51360–70. doi: 10.1021/acsomega.6c04466 (PMC13625119; doi:10.1021/acsomega.6c04466)
Supplement: Supplementary file 1 [file ao6c04466_si_001.pdf]

SUPPORTING INFORMATION

Thermodynamic stability of the icosahedral quasicrystal i-ScZn: A reassessment of the Sc–Zn phase diagram

Michael Sannemo-Targama,[1] Yu-Chin Huang,[2] Girma Gebresenbut,[2] Akash Khansili,[3] Roman Lucrezi,[1] Ulrich Häussermann[1,*]

[1]*Department of Chemistry, Stockholm University, 106 91 Stockholm, Sweden*

[2]*Department of Chemistry, Ångström Laboratory, Uppsala University, 751 21 Uppsala, Sweden*

[3]*Department of Physics, AlbaNova University Center, Stockholm University, 106 91 Stockholm, Sweden*

*email: Ulrich.Haussermann@su.se

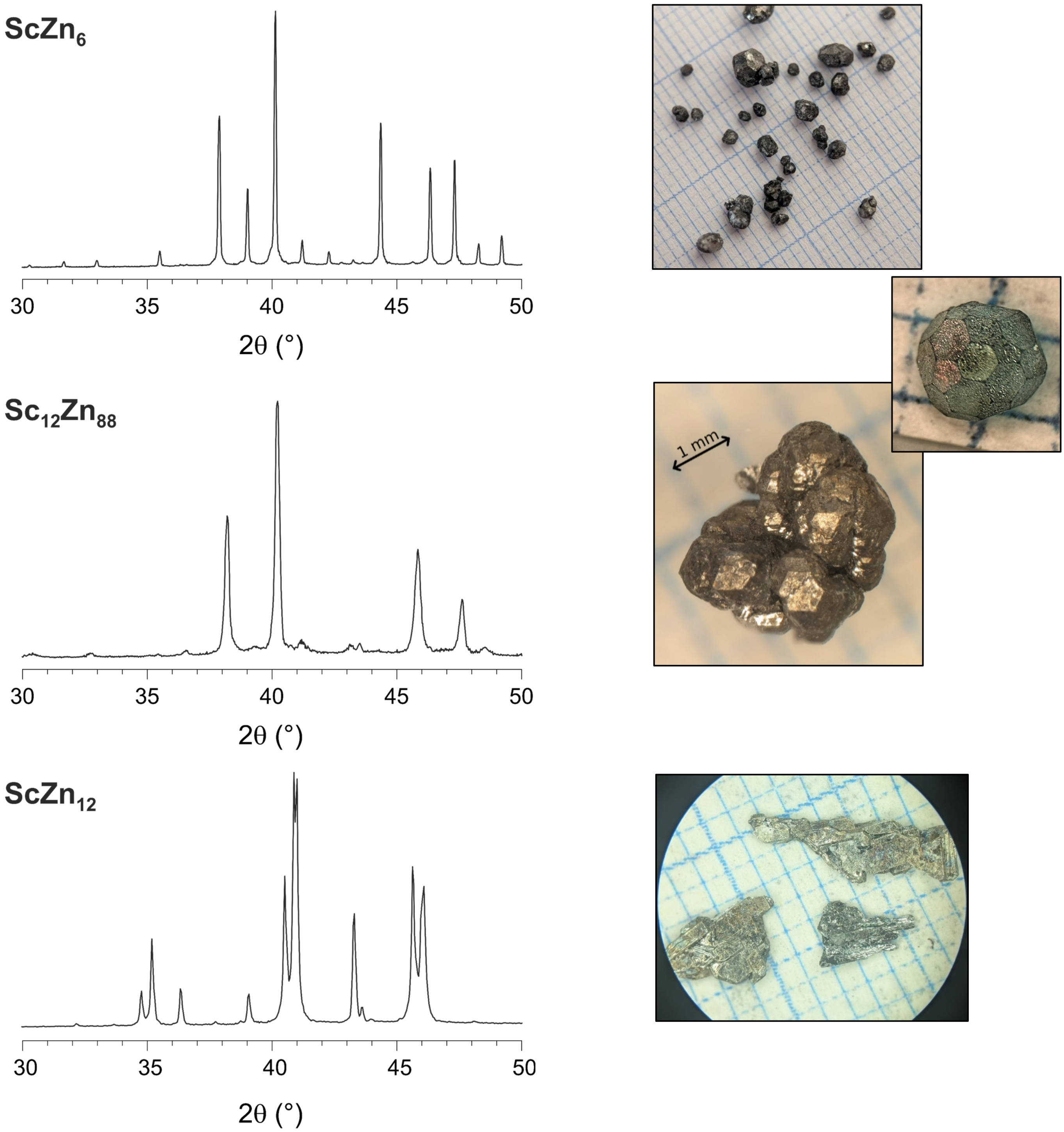

Fig.S1. PXRD patterns and photographs of as-cast specimens

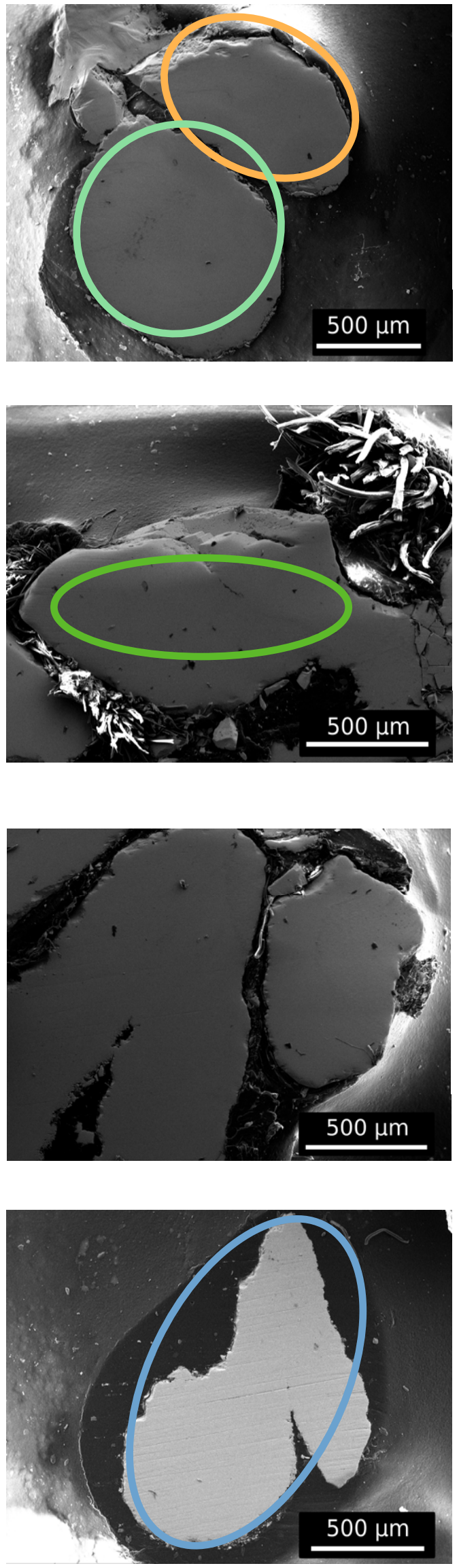

STATISTICS A	Sc	Zn
High	12.52%	88.14%
Low	11.86%	87.48%
Q1	12.11%	87.56%
Q3	12.44%	87.89%
IQR	0.33%	0.33%
Low Bound	11.62%	87.07%
High Bound	12.93%	88.38%
Average	12.25%	87.75%
Std. dev	**0.21%**	**0.21%**

STATISTICS B	Sc	Zn
High	12.48%	88.10%
Low	11.90%	87.52%
Q1	12.19%	87.57%
Q3	12.43%	87.81%
IQR	0.24%	0.24%
Low Bound	11.82%	87.21%
High Bound	12.79%	88.18%
Average	12.13%	87.87%
Std. dev	**0.26%**	**0.26%**

STATISTICS C	Sc	Zn
High	12.48%	88.10%
Low	11.90%	87.52%
Q1	12.19%	87.57%
Q3	12.43%	87.81%
IQR	0.24%	0.24%
Low Bound	11.82%	87.21%
High Bound	12.79%	88.18%
Average	12.28%	87.72%
Std. dev	**0.18%**	**0.18%**

STATISTICS D	Sc	Zn
High	12.59%	88.04%
Low	11.96%	87.41%
Q1	12.07%	87.67%
Q3	12.33%	87.93%
IQR	0.26%	0.26%
Low Bound	11.68%	87.28%
High Bound	12.72%	88.32%
Average	12.21%	87.79%
Std. dev	**0.18%**	**0.18%**

STATISTICS E	Sc	Zn
High	12.48%	92.17%
Low	7.83%	87.52%
Q1	11.89%	87.72%
Q3	12.29%	88.11%
IQR	0.40%	0.40%
Low Bound	11.29%	87.12%
High Bound	12.88%	88.71%
Average	12.21%	87.79%
Std. dev	**0.21%**	**0.21%**

STATISTICS F	Sc	Zn
High	12.47%	89.91%
Low	10.09%	87.53%
Q1	11.21%	87.71%
Q3	12.29%	88.79%
IQR	1.08%	1.08%
Low Bound	9.60%	86.10%
High Bound	13.90%	90.40%
Average	11.61%	88.39%
Std. dev	**0.88%**	**0.88%**

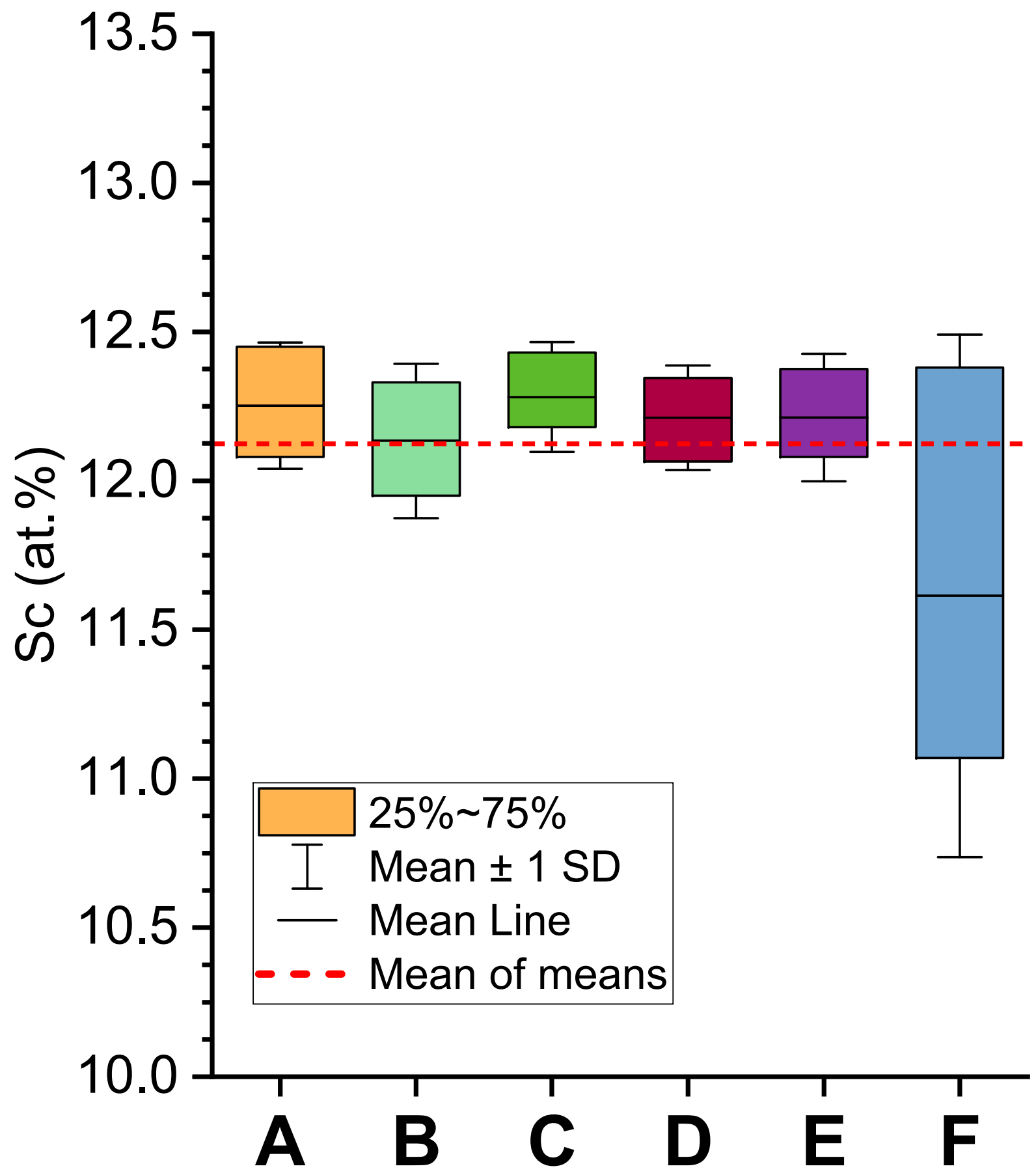

Fig. S2. EDS analysis of "as cast" $Sc_{12}Zn_{88}$ obtained from solution growth and centrifugation at 490 °C. Samples were prepared by cross section polishing. The large spread in F is attributed to the presence of $ScZn_{12}$.

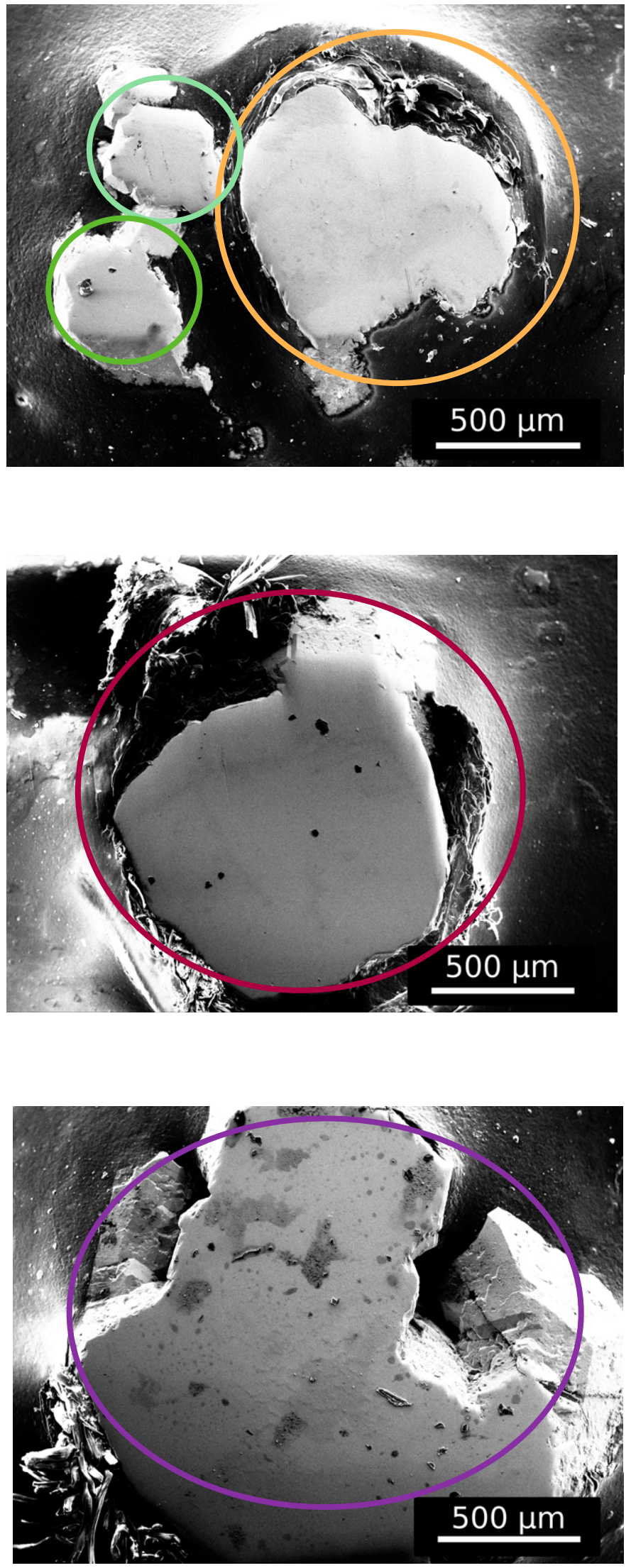

STATISTICS A	Sc	Zn
High	12.65%	88.03%
Low	11.97%	87.35%
Q1	12.18%	87.57%
Q3	12.43%	87.83%
IQR	0.26%	0.26%
Low Bound	11.79%	87.18%
High Bound	12.82%	88.21%
Average	11.96%	88.04%
Std. dev	0.22%	0.22%

STATISTICS B	Sc	Zn
High	12.55%	92.42%
Low	7.58%	87.45%
Q1	11.80%	87.69%
Q3	12.31%	88.21%
IQR	0.51%	0.51%
Low Bound	11.03%	86.92%
High Bound	13.08%	88.97%
Average	11.80%	88.20%
Std. dev	1.47%	1.47%

STATISTICS C	Sc	Zn
High	12.18%	90.88%
Low	9.12%	87.82%
Q1	11.86%	87.90%
Q3	12.10%	88.14%
IQR	0.24%	0.24%
Low Bound	11.50%	87.54%
High Bound	12.46%	88.50%
Average	12.08%	87.92%
Std. dev	1.12%	1.12%

STATISTICS D	Sc	Zn
High	12.49%	87.84%
Low	12.16%	87.51%
Q1	12.23%	87.64%
Q3	12.36%	87.77%
IQR	0.14%	0.13%
Low Bound	12.03%	87.44%
High Bound	12.57%	87.98%
Average	12.23%	87.77%
Std. dev	**0.10%**	**0.10%**

STATISTICS E	Sc	Zn
High	12.49%	88.08%
Low	11.92%	87.51%
Q1	12.12%	87.57%
Q3	12.43%	87.88%
IQR	0.31%	0.31%
Low Bound	11.66%	87.11%
High Bound	12.89%	88.34%
Average	12.15%	87.85%
Std. dev	0.20%	0.20%

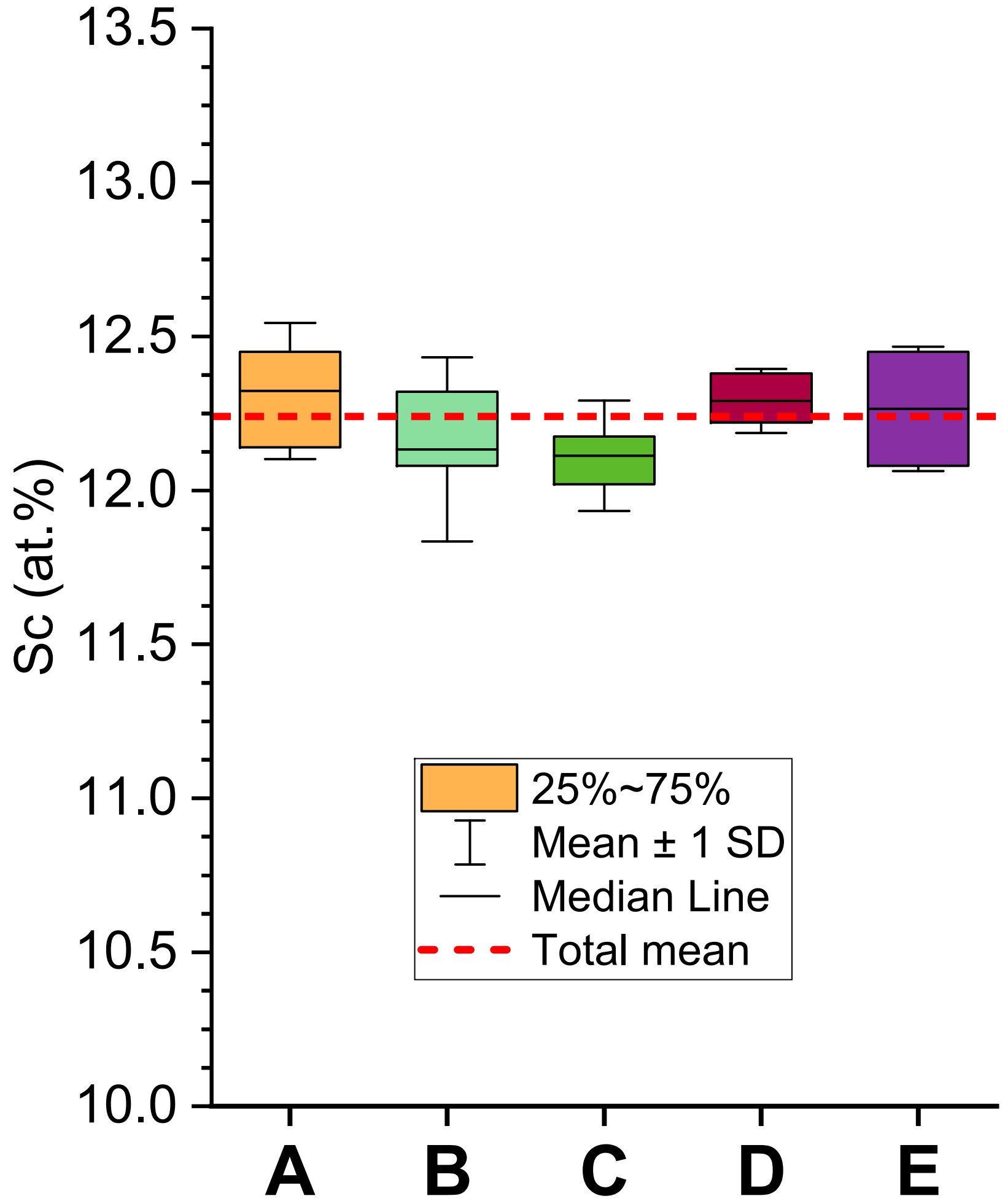

Fig. S3. EDS analysis of "as cast" $Sc_{12}Zn_{88}$ obtained from solution growth and centrifugation at 510 °C. Samples were prepared by cross section polishing.

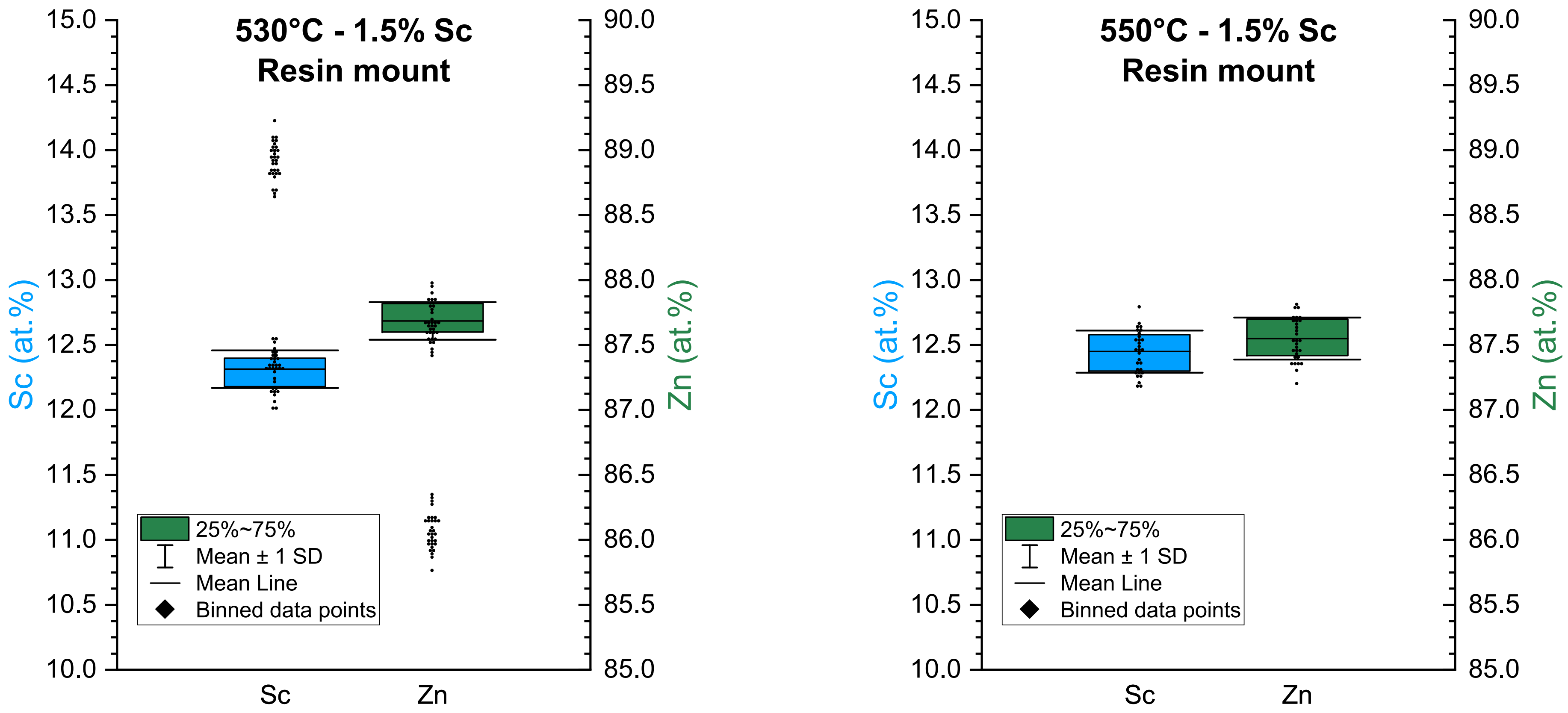

Fig. S4. EDS analysis of "as cast" $Sc_{12}Zn_{88}$ obtained from solution growth and centrifugation at 530 °C (left) and 550 °C (right). Samples corresponded to resin mounted powders. Data points in the 530 °C sample clearly have a bimodal distribution around compositions corresponding to QC and 1/1 AC. For statistical calculations, the clustered points around Sc ≈ 14 at.% and Zn ≈ 86 at.% were excluded.

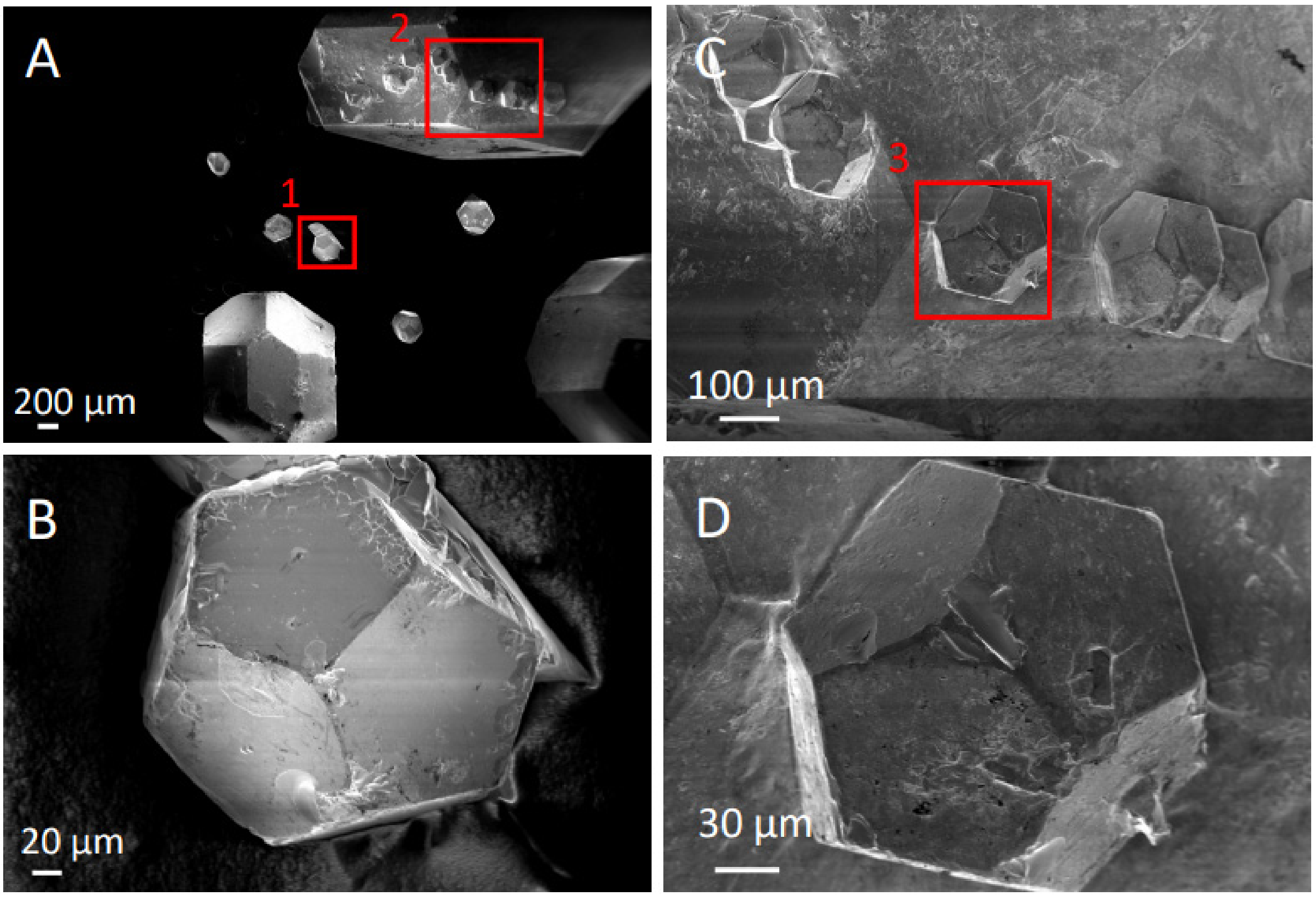

Fig. S5. A) Cooling a melt $Sc_{7.7}Zn_{92.3}$ ($ScZn_{12}$) from 800 to 490 °C and subsequent centrifugation produces large, mm-sized, $ScZn_6$ AC crystals and traces of QC phase. Fig.s b) and c) correspond to sections 1 and 2, respectively, marked in Fig. a). Fig. d) corresponds to section 3 marked in Fig. c). SEM images were acquired with a Zeiss-Merlin instrument.

.

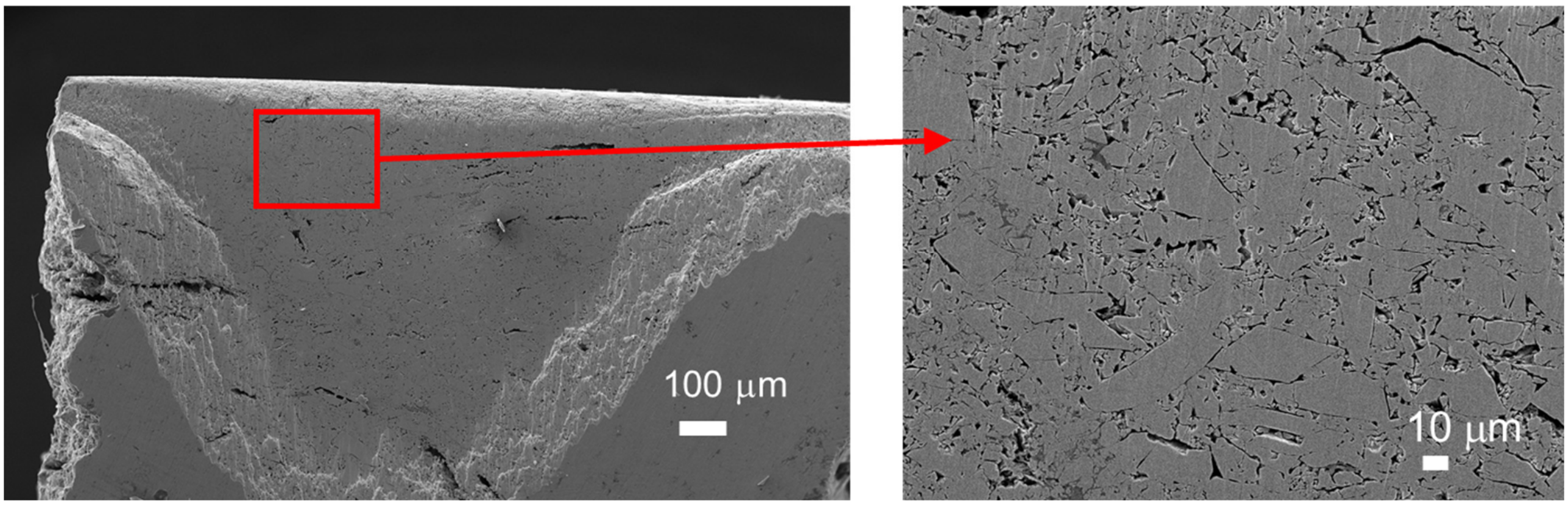

STATISTICS A	Sc	Zn
High	13.20%	91.80%
Low	8.20%	86.80%
Q1	12.16%	87.45%
Q3	12.55%	87.84%
IQR	0.39%	0.39%
Low Bound	11.58%	86.87%
High Bound	13.13%	88.42%
Average	12.45%	87.55%
Std. dev	**0.15%**	**0.15%**

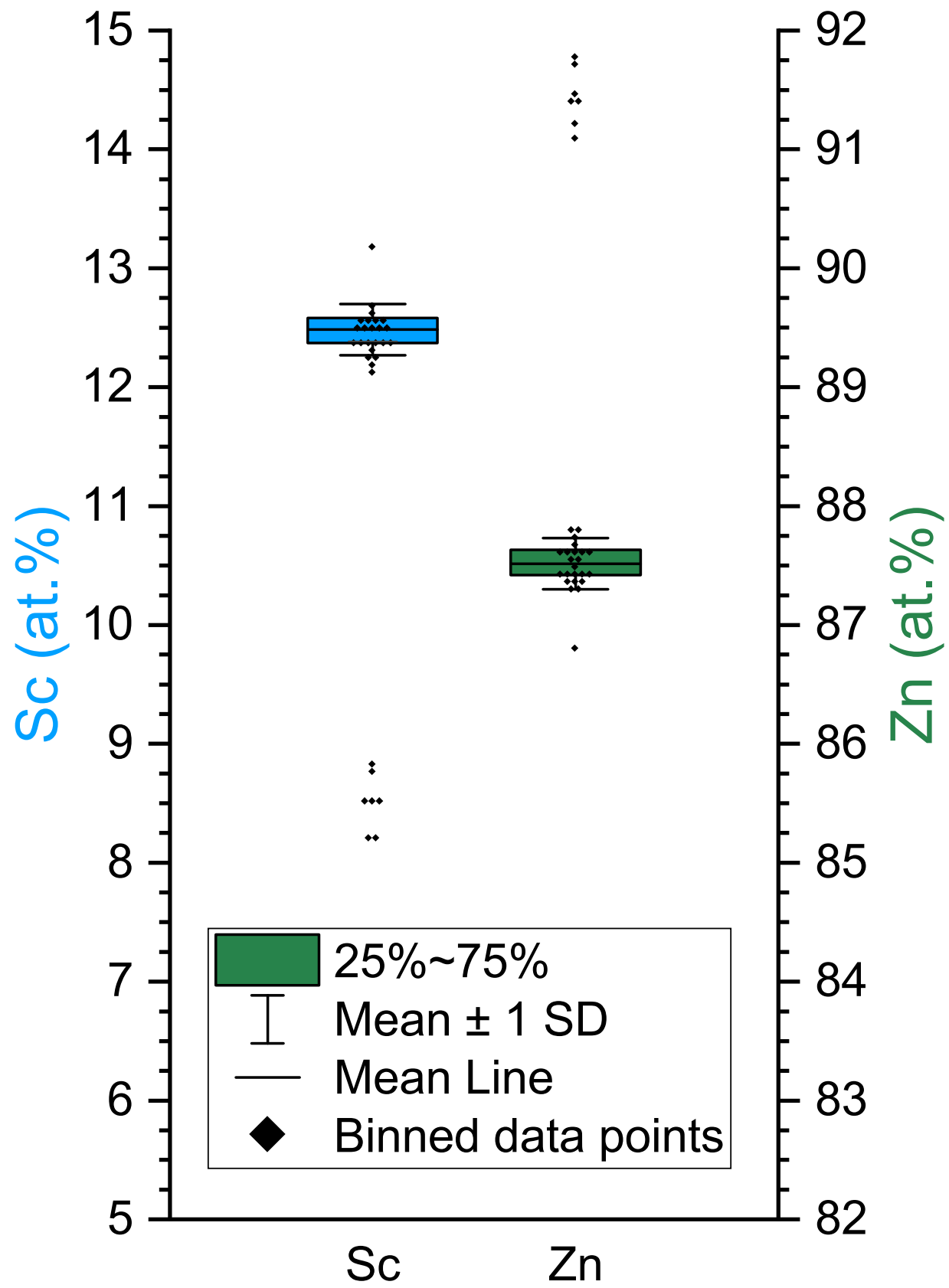

Fig. S6. EDS analysis of the product from the peritectoid reaction 3.5 $ScZn_6$ + $ScZn_{12}$ at 490 °C and 48 h duration. The sample corresponded to a cross section polished fragment of the reaction pellet.

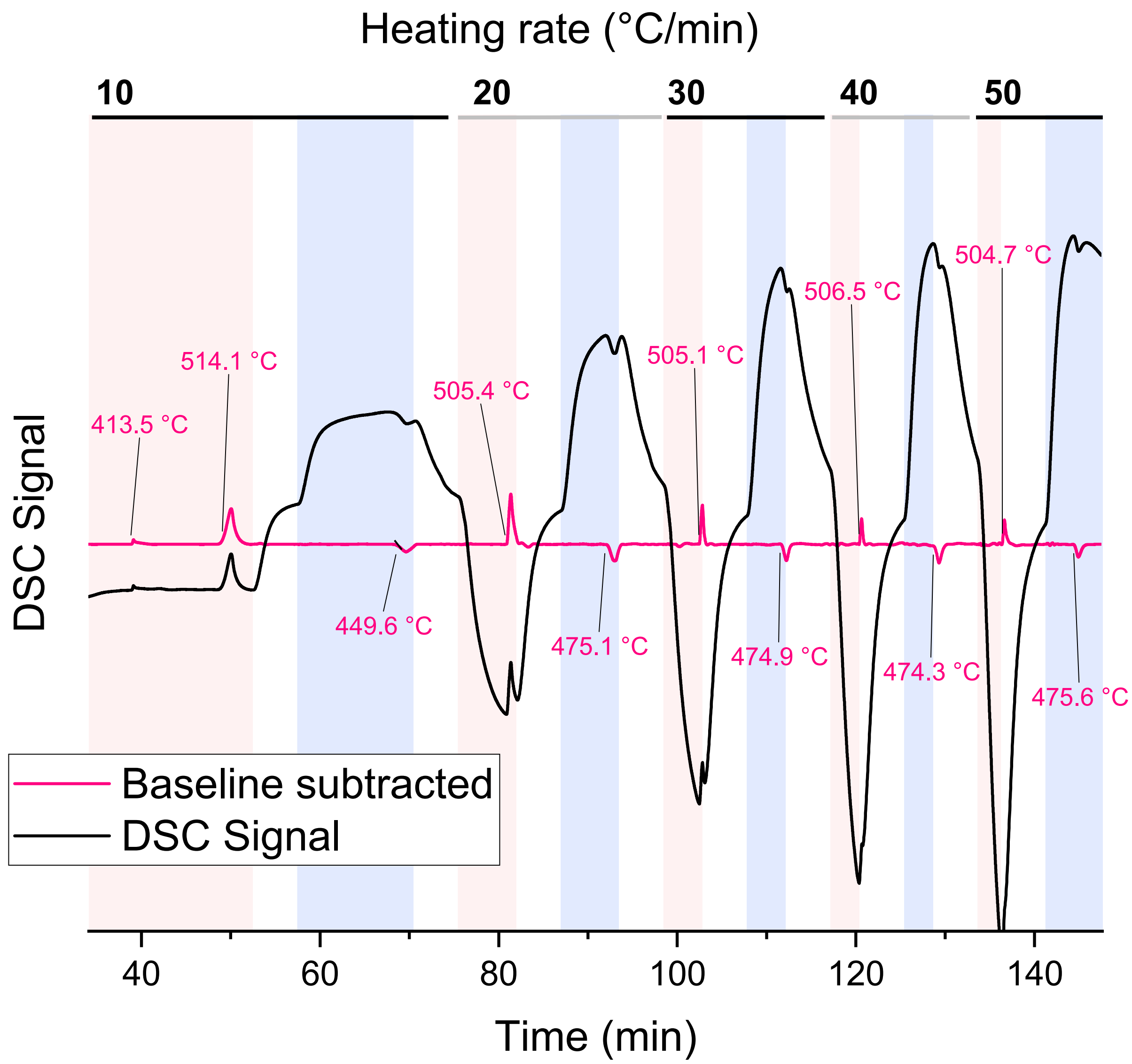

Fig. S7. DSC raw and background-subtracted signal of a piece of $ScZn_{12}$ (18.2 mg) during repeated heating and cooling (between 400 and 550 °C), with the heating/cooling rate increased by 10 °C/min each cycle, from 10 to 50 °C/min. Red regions are heating, blue cooling, white isothermal. Due to the large mass of the crucible (stainless steel, ~1000 mg), thermal inertia is observed (i.e. there is a delay between the nominal and actual heating rate), which is why for the higher heating rates the signals do not appear until the isothermal region.

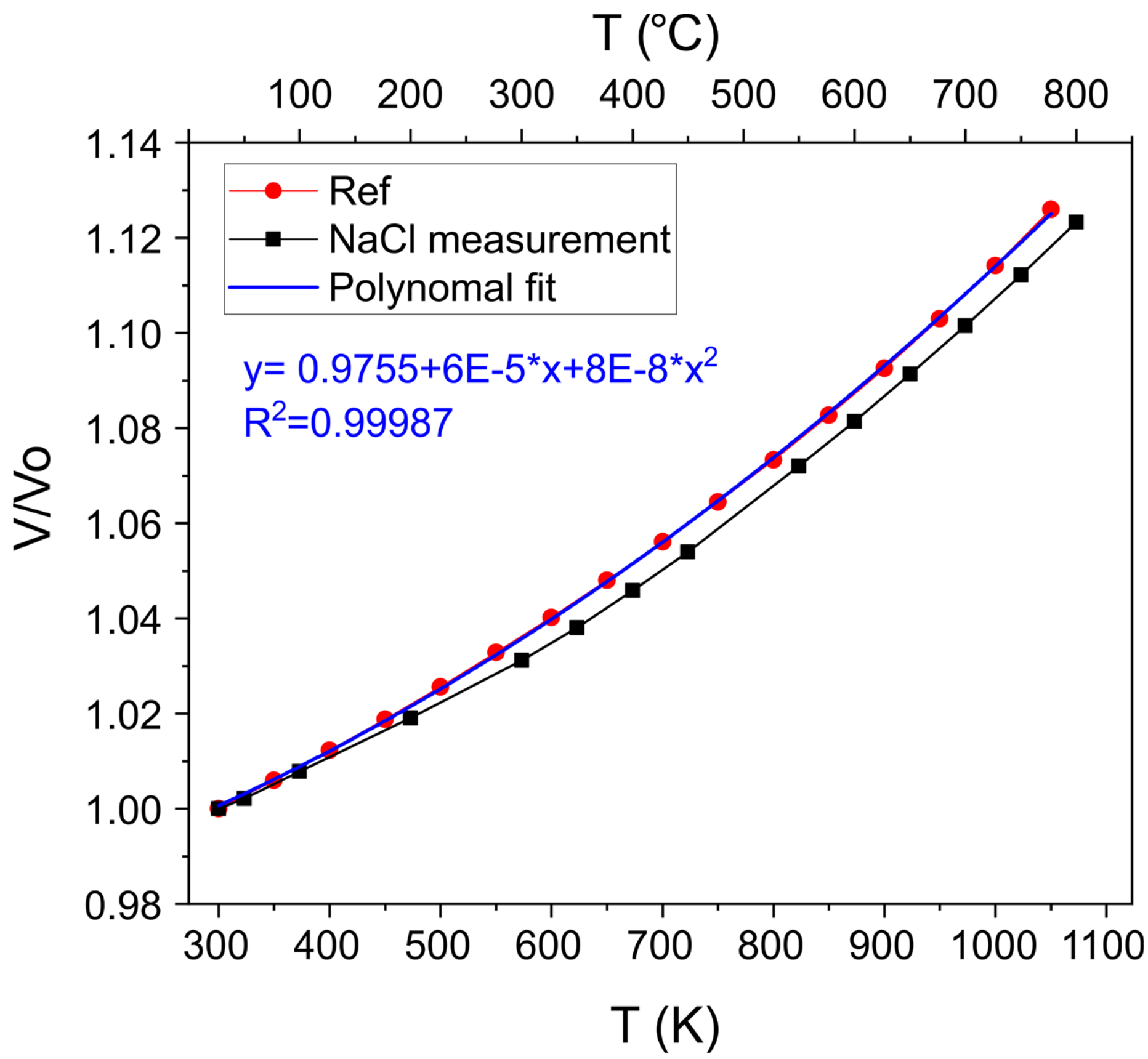

T(K) measured	T(K) NaCl calibrated
300	293.5
323	313.5
373	363.3
473	452.6
573	539.3
623	585.7
673	634.8
723	684.0
823	785.6
873	834.9
923	885.6
973	934.6
1023	984.7
1073	1034.7

Fig. S8. Temperature calibration for the variable *T* PXRD measurements using NaCl as calibrant, see Fang, Z.-H. Temperature Dependence of Volume Thermal Expansion for NaCl and KCl Crystals. *Physica B: Condensed Matter* **2005**, *357* (3), 433–438.

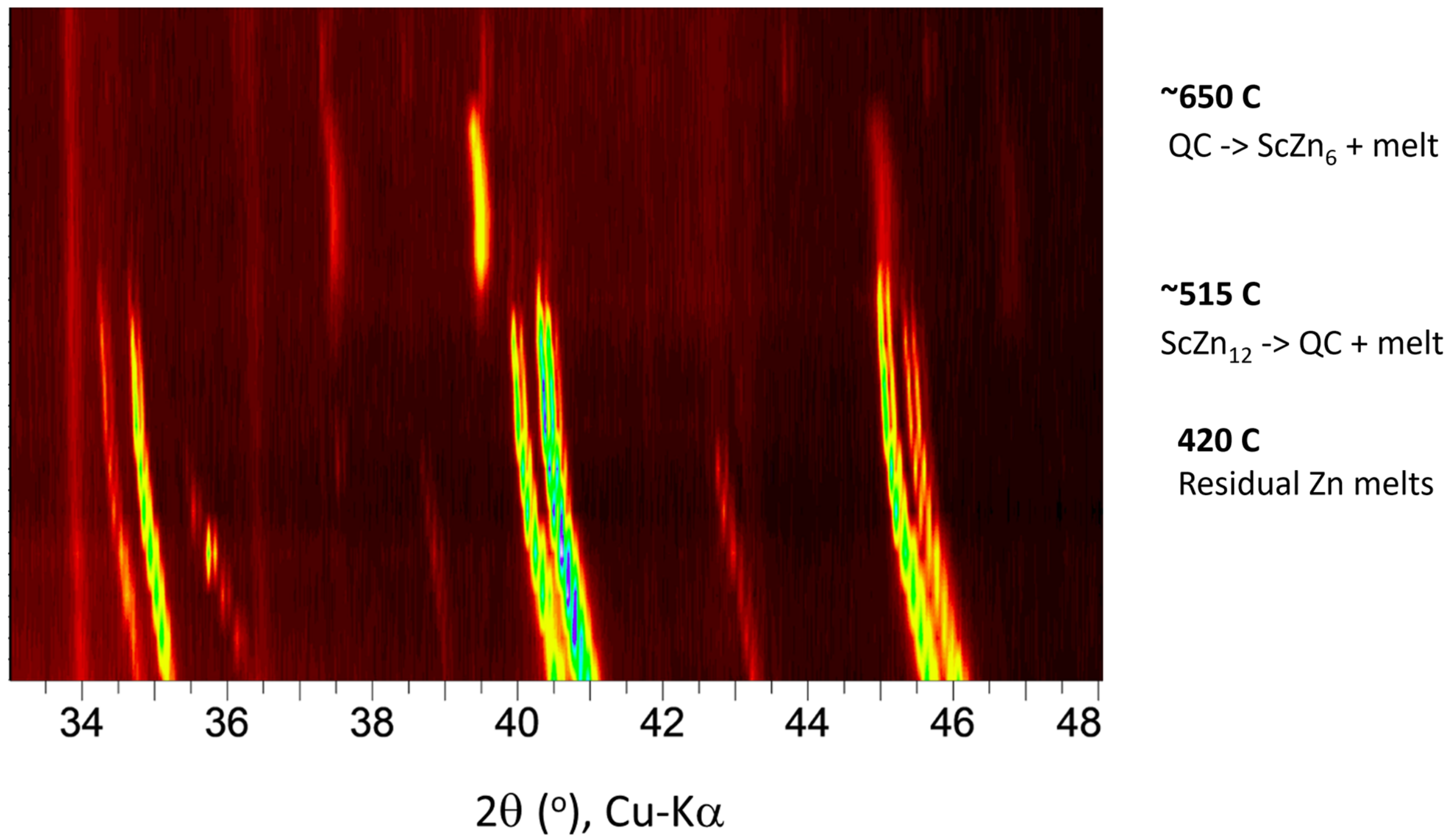

Fig. S9. Heat map presentation of the patterns obtained from variable-*T* PXRD measurement of $ScZn_{12}$ (cf. Fig.6a). 1

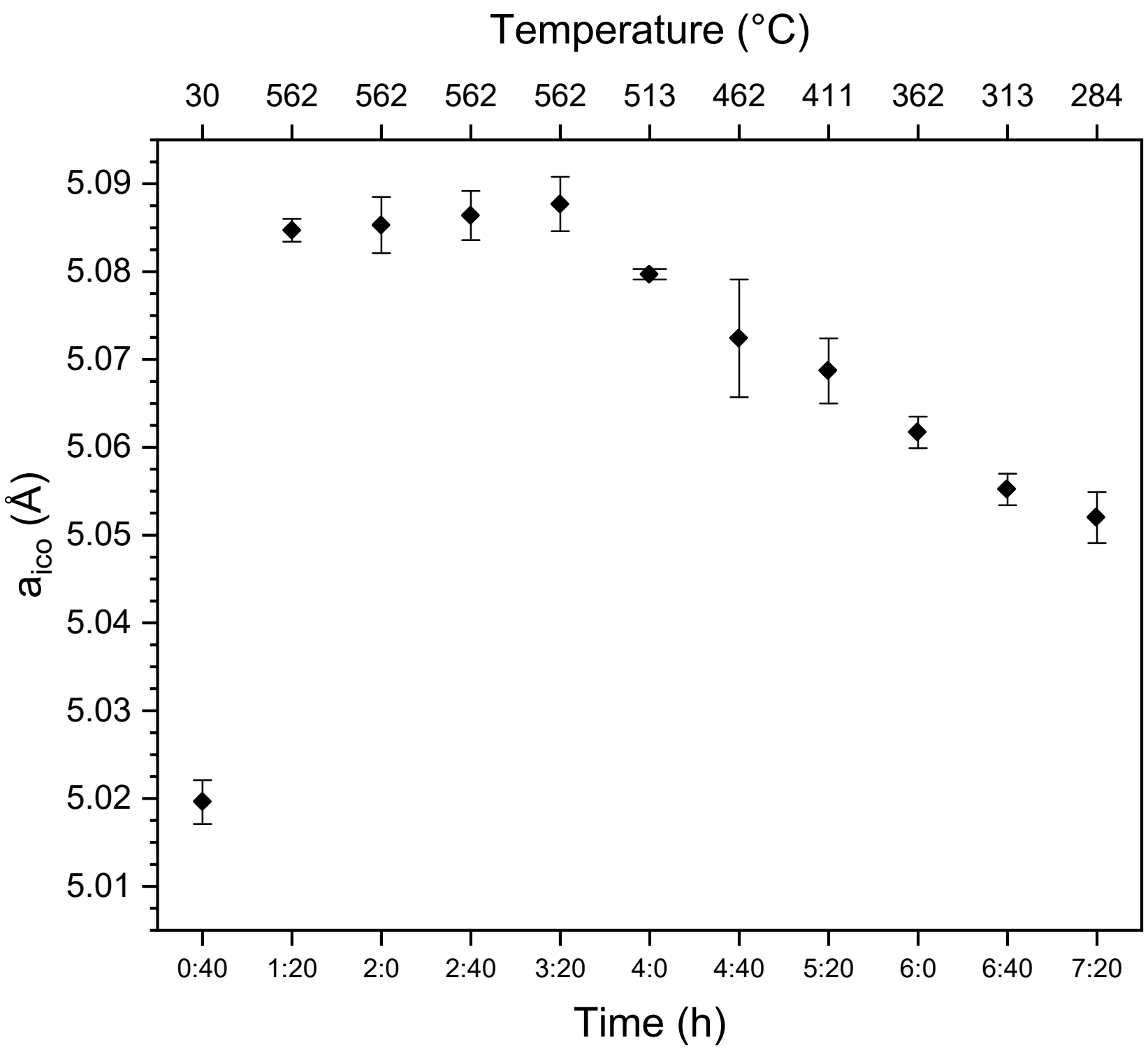

Fig. S10. Variation of the quasilattice parameter for i-ScZn across the experiment shown in Fig.7: Rapid heating of i-ScZn from room temperature to 562 °C is followed by annealing for 4 h and subsequent slow cooling to 285 °C (ca 20 °C/h).

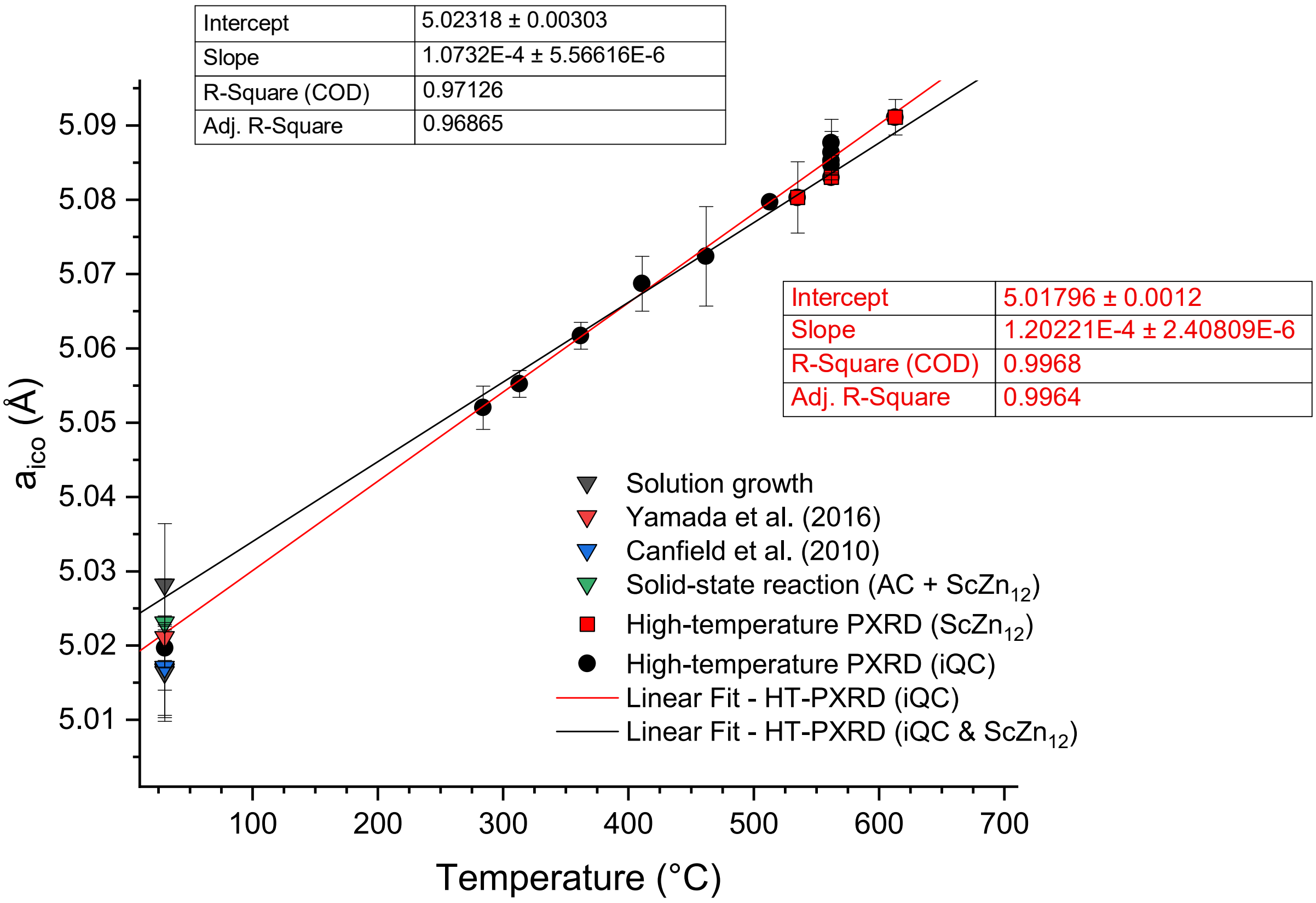

Temperature (°C)	Quasilattice parameter a_{ico} (Å)
**284**	5.052(3)
**313**	5.055(2)
**362**	5.062(2)
**411**	5.069(4)
**462**	5.072(7)
**513**	5.0797(6)
**562**	5.088(3)
**562**	5.086(3)
**562**	5.085(3)
**562**	5.085(1)
**535**	5.020(3)
**562**	5.0830(3)
**613**	5.091(2)

Fig. S11. Temperature-dependence of the quasilattice parameter of i-ScZn in the temperature range 300 – 600 °C (two experiments, black circles and red squares (cf. Figs. 7 and 6a, respectively). The linear fits refer either to the black circle data points only (black line) or to both black circles and red squares (red line). Room-temperature data of various i-ScZn samples are included according to Table 1.

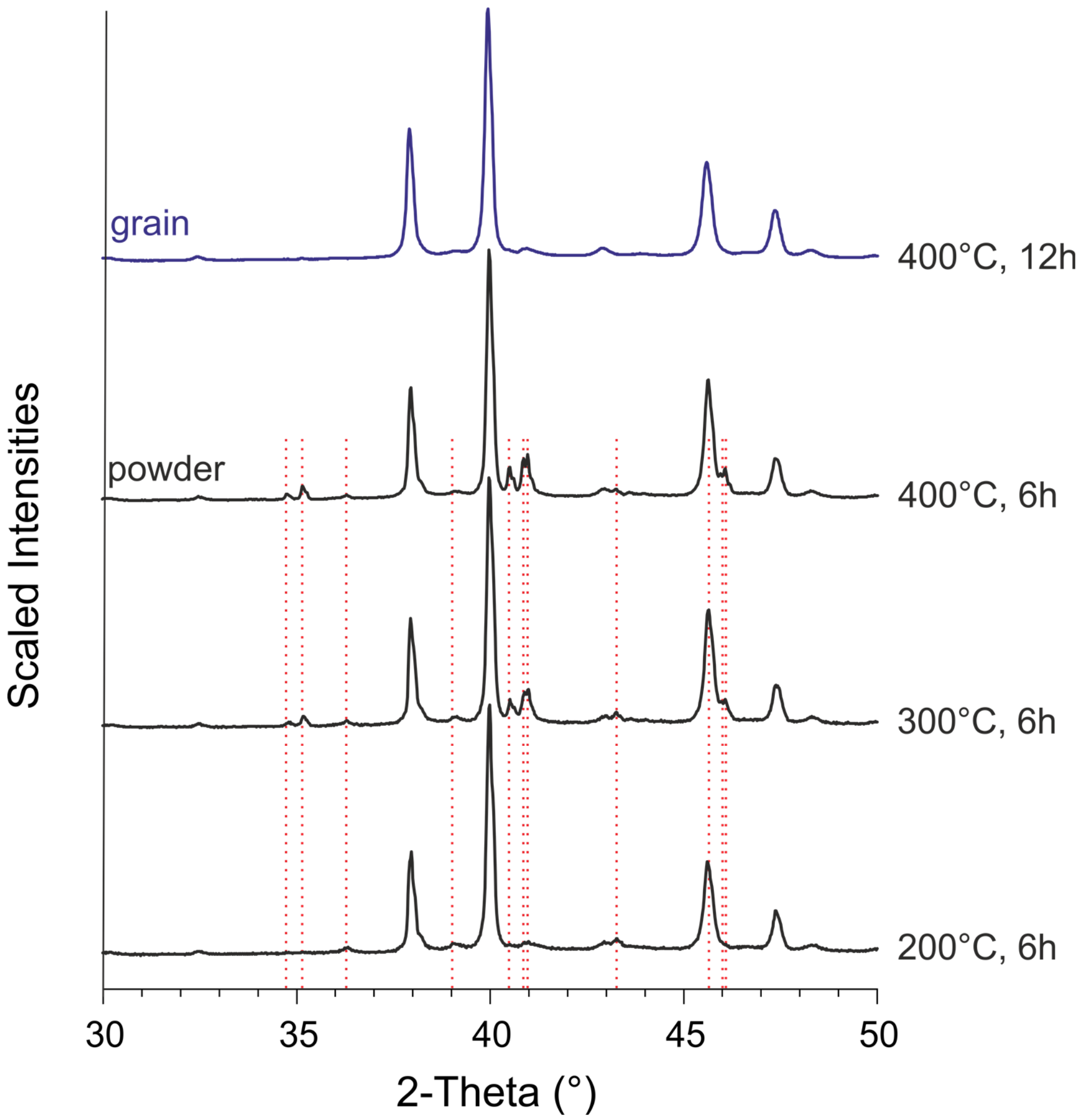

Fig. S12. Annealing results for $Sc_{12}Zn_{88}$. Reflection positions of $ScZn_{12}$ are marked as dotted red lines. (Note that the grain specimen was ground to a powder after the annealing experiments). The quasilattice parameters for the annealed powders are 200 °C: 5.020(8) Å, 300 °C: 5.020(3) Å, 400 °C: 5.022(3) Å and for the annealed grain 400 °C: 5.022(3) Å.

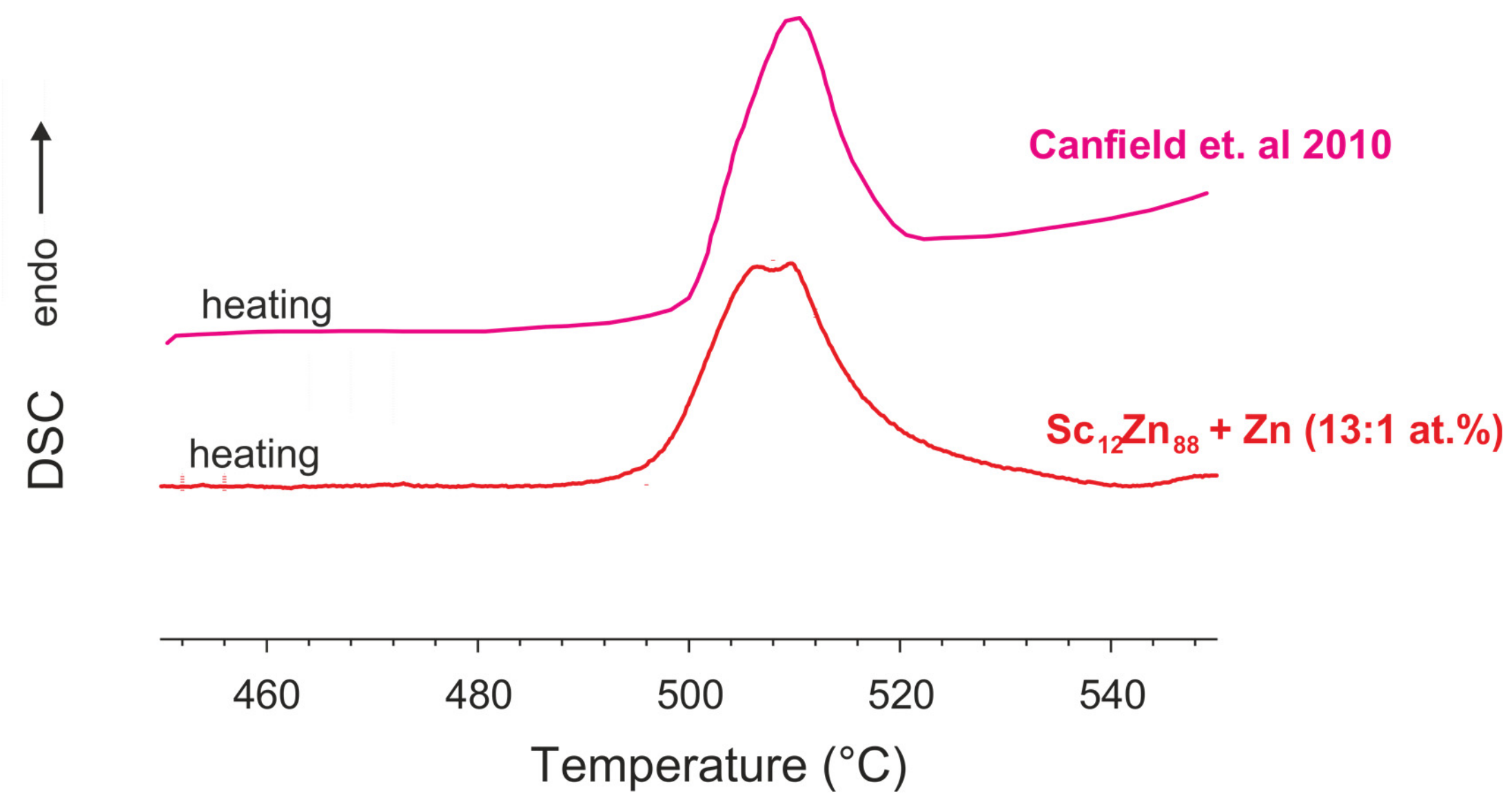

Fig. S13. DSC trace of a mixture of "as cast" $Sn_{12}Zn_{88}$ and elemental Zn (13:1 at.%) upon heating in comparison with the reported DSC trace of $Sn_{12}Zn_{88}$ by Canfield, P. C.; Caudle, M. L.; Ho, C.-S.; Kreyssig, A.; Nandi, S.; Kim, M. G.; Lin, X.; Kracher, A.; Dennis, K. W.; McCallum, R. W.; Goldman, A. I. Solution Growth of a Binary Icosahedral Quasicrystal of Sc 12 Zn 88. *Phys. Rev. B* **2010**, *81* (2), 020201

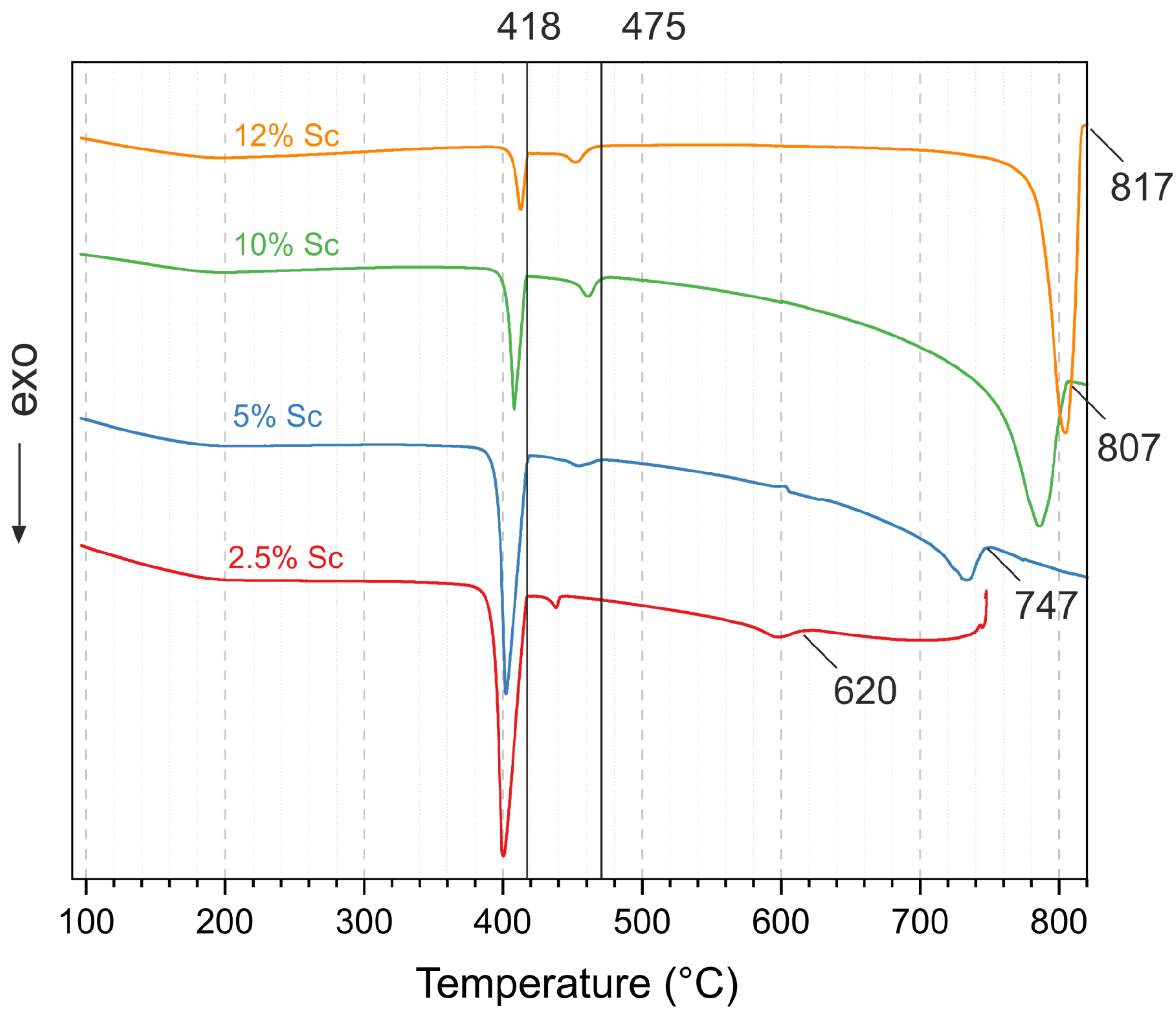

Fig. S14. DSC traces of reactions mixture 12, 10, 5, 2.5 at.% Sc upon cooling from 850 °C (750 °C for 2.5%). The trace for 2.5 at.% Sc is suspected to display supercooling since the crossing of the liquidus line is expected around 700 °C.

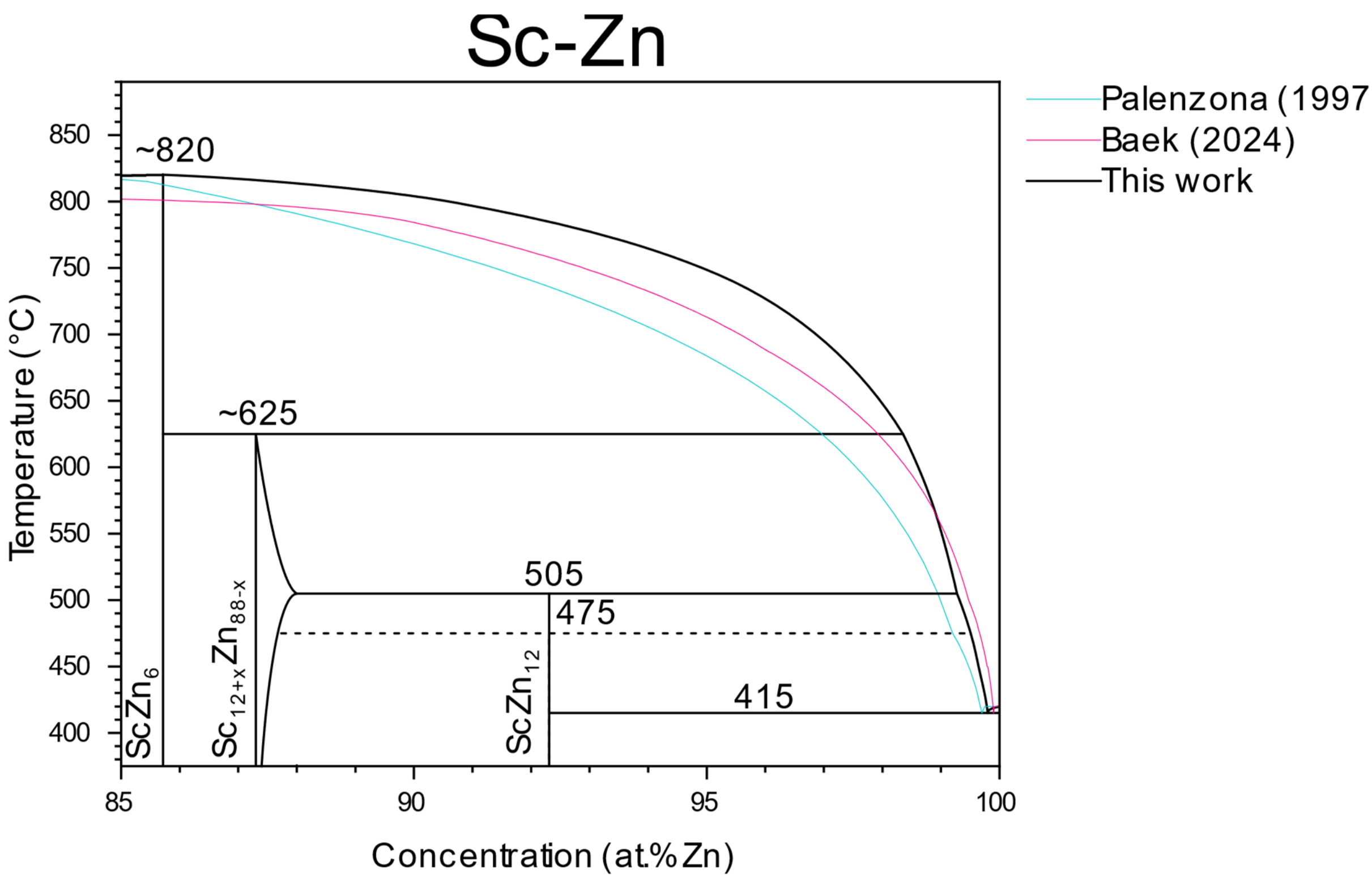

Fig. S15. Comparison of liquidus lines for the Zn-rich part of the Sc-Zn phase diagram. Palenzona (1997): Palenzona, A.; Manfrinetti, P. The Phase Diagram of the Sc-Zn System. *Journal of Alloys and Compounds* **1997**, *247* (1), 195–197. Baek (2024): Baek, W.; Das, S.; Tan, S.; Gavini, V.; Sun, W. Quasicrystal Stability and Nucleation Kinetics from Density Functional Theory. *Nat. Phys.* **2025**, *21* (6), 980–987.

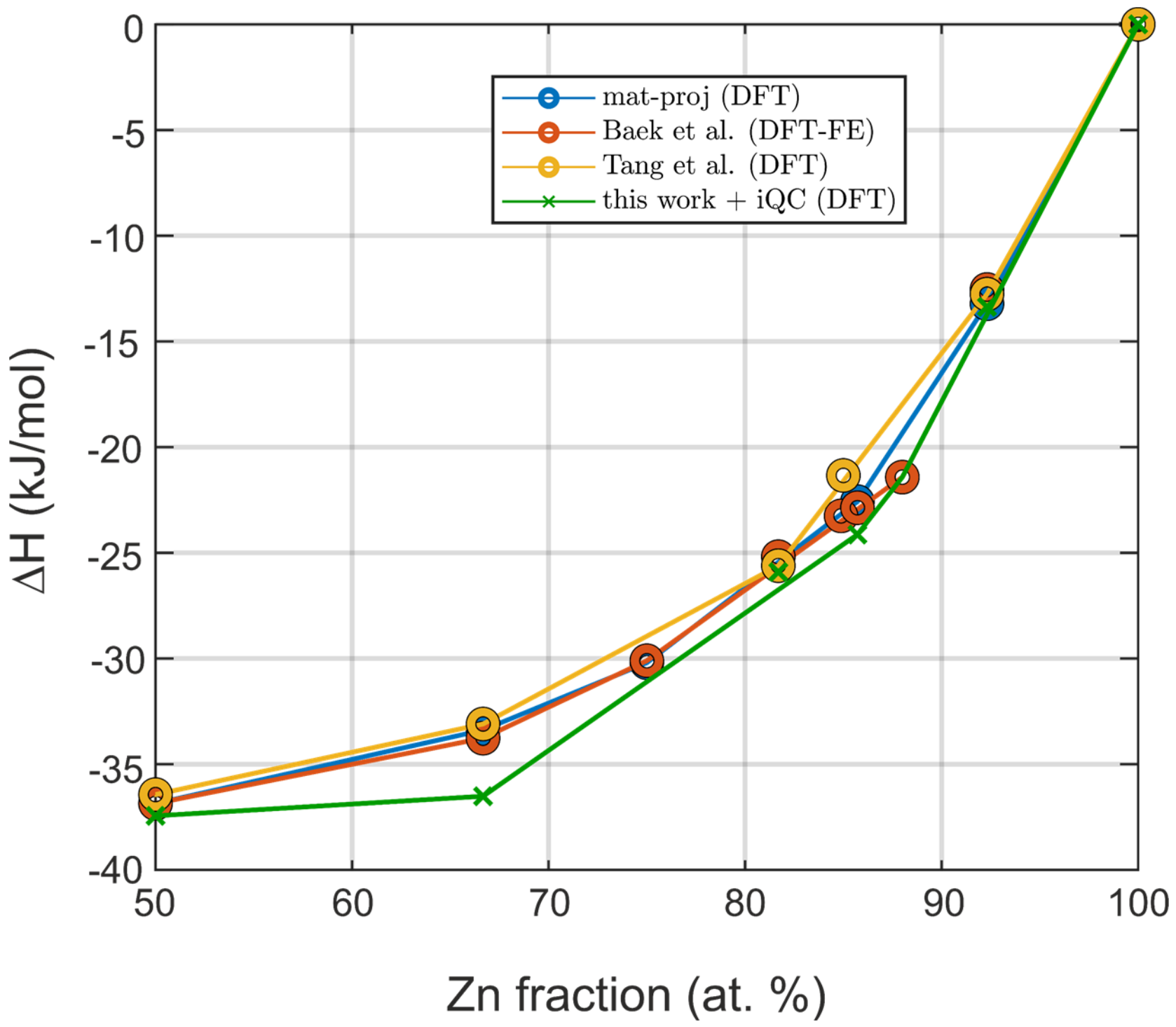

Fig.S16. Extended $T = 0$ K convex hull considering all known Sc-Zn phases. Note that $ScZn_2$ crystallizes with the hexagonal AlB_2 structure, which is substantially more stable than the hexagonal Laves phase structure (C14) assumed in previous works. $ScZn_3$ does not exist. Hexagonal $Sc_{13}Zn_{58}$ (81.7 at.% Zn, $Gd_{13}Zn_{58}$ structure) is slightly above the hull. The previously assumed composition Sc_3Zn_{17} (85 at.% Zn) for the 1/1 AC phase ($ScZn_6$) is clearly unstable. As a reference, the enthalpy of formation was determined experimentally for ScZn by drop solution calorimetry. The value (referring to 298 K) is -41.4 ± 3.0 kJ/mol atom, taken from Meschel, S. V.; Nash, P. Thermochemistry of Some Zinc-Transition Metal(TM) Compounds and Some Bismuth-TM Compounds by High Temperature Direct Synthesis Calorimetry. *Calphad* **2019**, *64*, 131–138.
